# Supplementary material for: The transcription factor STAT5 catalyzes Mannich ligation reactions yielding inhibitors of leukemic cell proliferation
Source: Nat Commun. 2019 Jan 8;10:66. doi: 10.1038/s41467-018-07923-2 (PMC6325109; doi:10.1038/s41467-018-07923-2)
Supplement: Supplementary file 1 — Supplementary Information [file 41467_2018_7923_MOESM1_ESM.pdf]

SUPPLEMENTARY INFORMATION

The transcription factor STAT5 catalyzes Mannich ligation reactions yielding inhibitors of leukemic cell proliferation

Wong et al.

Supplementary Figures

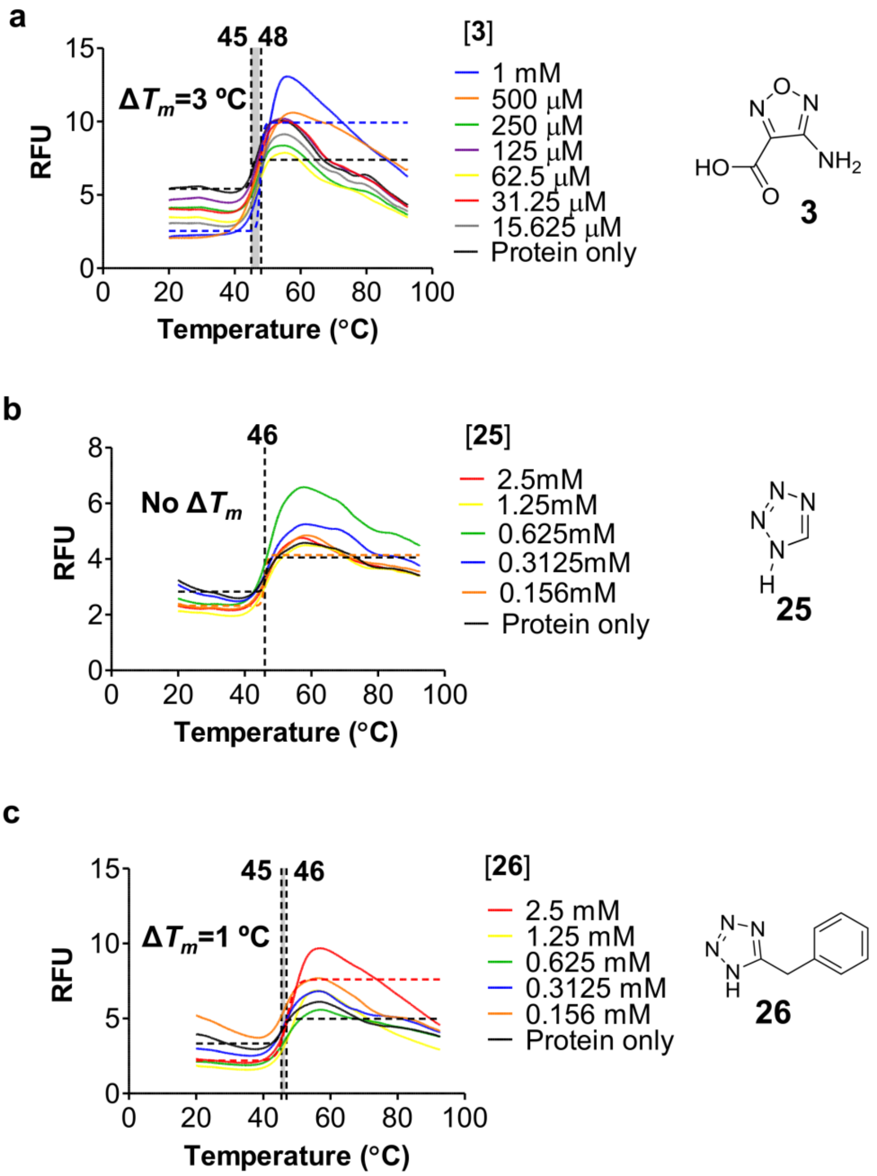

Supplementary Figure 1. Thermal shift assays of fragments 3, 25 and 26 with MBP-STAT5b. a, Fragment **3** induced a significant shift in the melting temperature (ΔT_m= 3 ºC) of MBP-STAT5b protein at 1 mM. **b-c**, Fragments **25** and **26**, respectively, show no or minute T_m shift (1 ºC) in the thermal denaturation curves of MBP-STAT5b. The fluorescence changes shown in the plot with increasing temperature increased were fitted to the Boltzmann equation by non-linear regression to obtain the melting temperature, T_m (data shown are one representative of n=3).

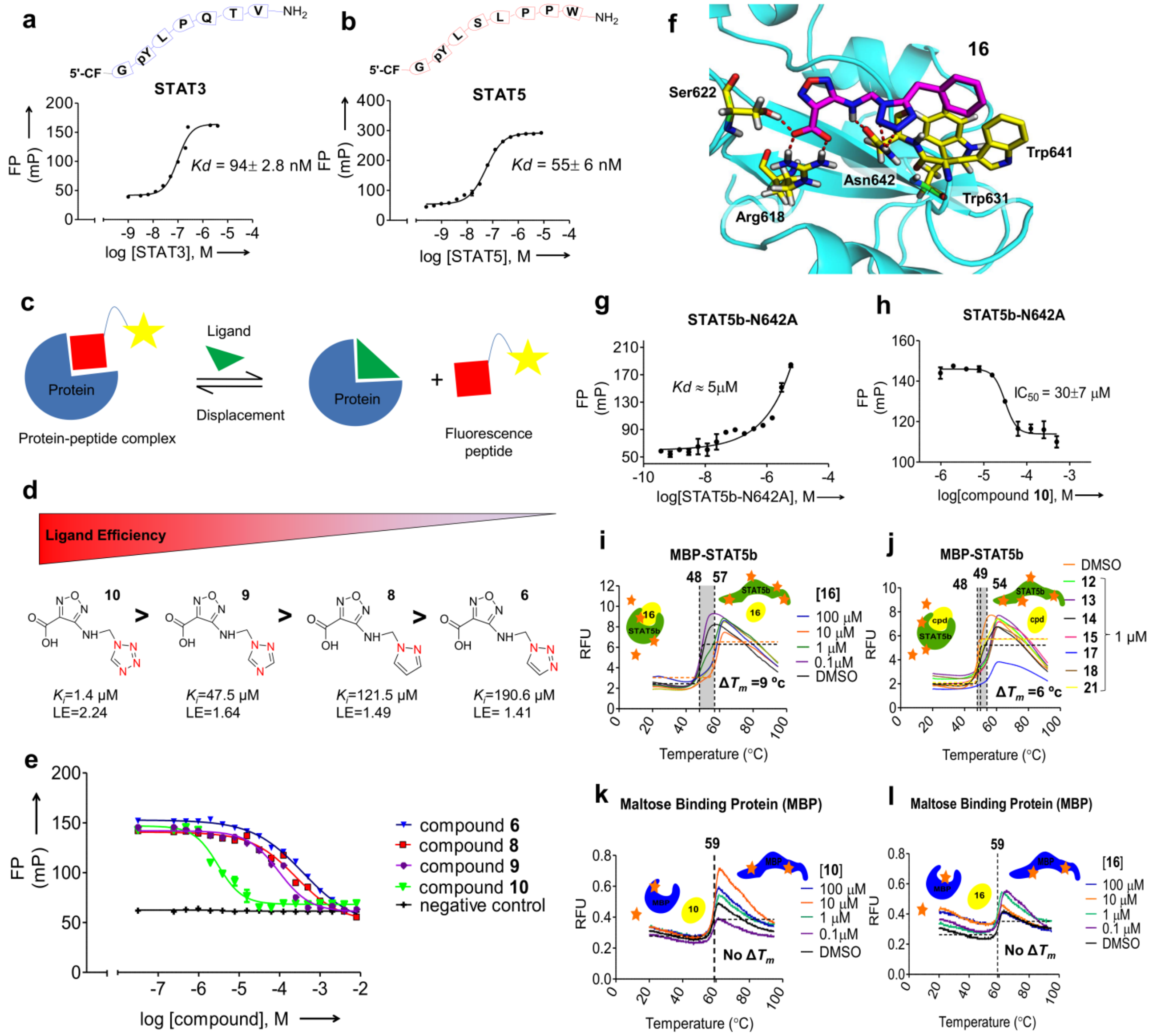

Supplementary Figure 2. Validation of Asn642 as key binding residue of STAT5-SH2 domain. a-b, Binding of carboxyfluorescein phosphopeptides 5-CF-GpYLPQTV-NH_2 to STAT3 and 5-CF-GpYLSLPPW-NH_2 (**1**) to STAT5b yielding K_D-values of 94 ±2.8 nM for STAT3 and 55 ±6 nM for STAT5b (*n*=3). **c**, Schematic illustration of the fluorescence polarization (FP) binding assay. **d**, K_I values and ligand efficiencies of compounds **6-10**. **e**, Binding of compounds **6**, **8**, **9**, and **10** to recombinant STAT5b (*n*=3). **f**, Binding mode of compound **16** to STAT5b-SH2 domain showing hydrogen bond formation with residue Asn642. **g-h**, Binding of phosphopeptide **1** and of compound **10** to mutant STAT5b-N642A (*n*=3). **i**, Compound **16** stabilized STAT5b protein shifting the melting temperature (ΔT_m) of MBP-STAT5b-SH2 protein by 9 ºC in the thermal shift assay (TSA) (data shown are one representative of *n*=3). **j**, Compounds **12-15**, **17**, **18**, and **21** induced significant shifts in the melting temperature of MBP-STAT5b-SH2 (ΔT_m= 6 ºC) at 1 µM (data shown are one representative of *n*=3). **k-l**, Compounds **10** and **16** did not bind to maltose binding protein (MBP). Error bars denote mean ± S.D. (*n*=3).

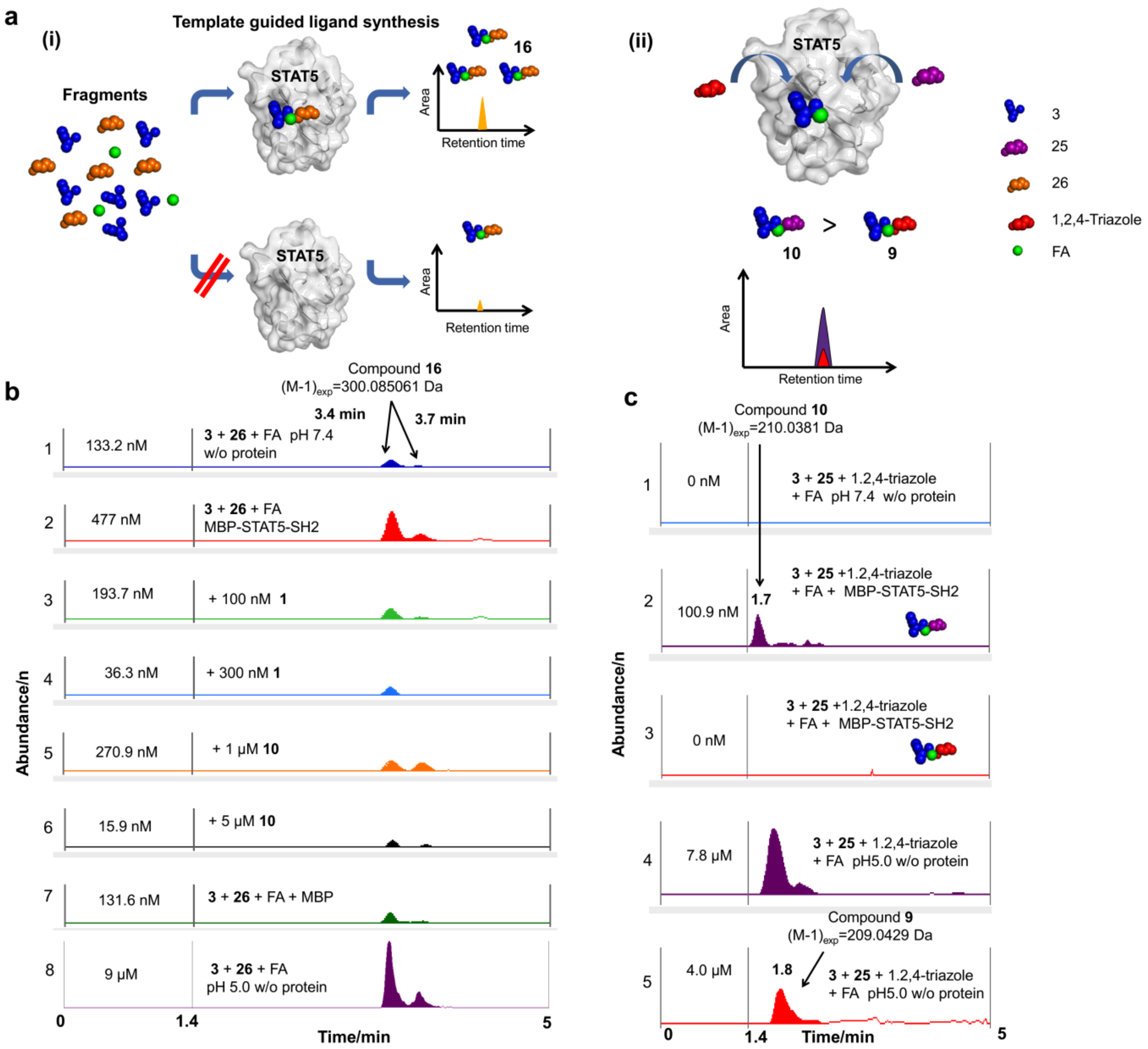

Supplementary Figure 3. Protein-induced formation of inhibitors through Mannich ligations. a (**i**), Schematic illustration of protein-induced formation of compound **16**. (**ii**) Formation of compounds **10** and **9** from a fragment mixture (**3**, **25**, and 1, 2, 4-triazole) incubated with FA in the presence of MBP-STAT5b-SH2. **b**, Formation of compound **16** detected in the HPLC-QTOF-MS. Lane 1: Negative control, formation of **16** from FA, fragments **3** and **26** at pH 7.4 in MOPS buffer without MBP-STAT5b-SH2. 2: Protein-induced formation of compound **16** at pH 7.4. Lanes 3-6: Inhibition of the protein-induced formation of compound **16** by peptide **1** (3,4) or inhibitor **10** (5,6). 7: Formation of compound **16** in the presence of Maltose Binding Protein. 8: Formation of compound **16** at pH 5.0 without protein (representative example of three independent experiments, *n*=3). **c**, Formation of compounds **9** and **10** detected by HPLC-QTOF-MS. Lane 1: negative control, no formation of **9** or **10** from FA, fragments **3, 25** and 1, 2, 4-triazole at pH 7.4 in MOPS buffer without MBP-STAT5b-SH2. 2: Formation of compound **10** with protein at pH 7.4. 3: No formation of compound **9** was detected with protein at pH 7.4. 4: Formation of compound **9** and **10** at pH 5.0 without protein; formation of compound **10** is 2x higher compared to **9** (representative example of three independent experiments, *n*=3).

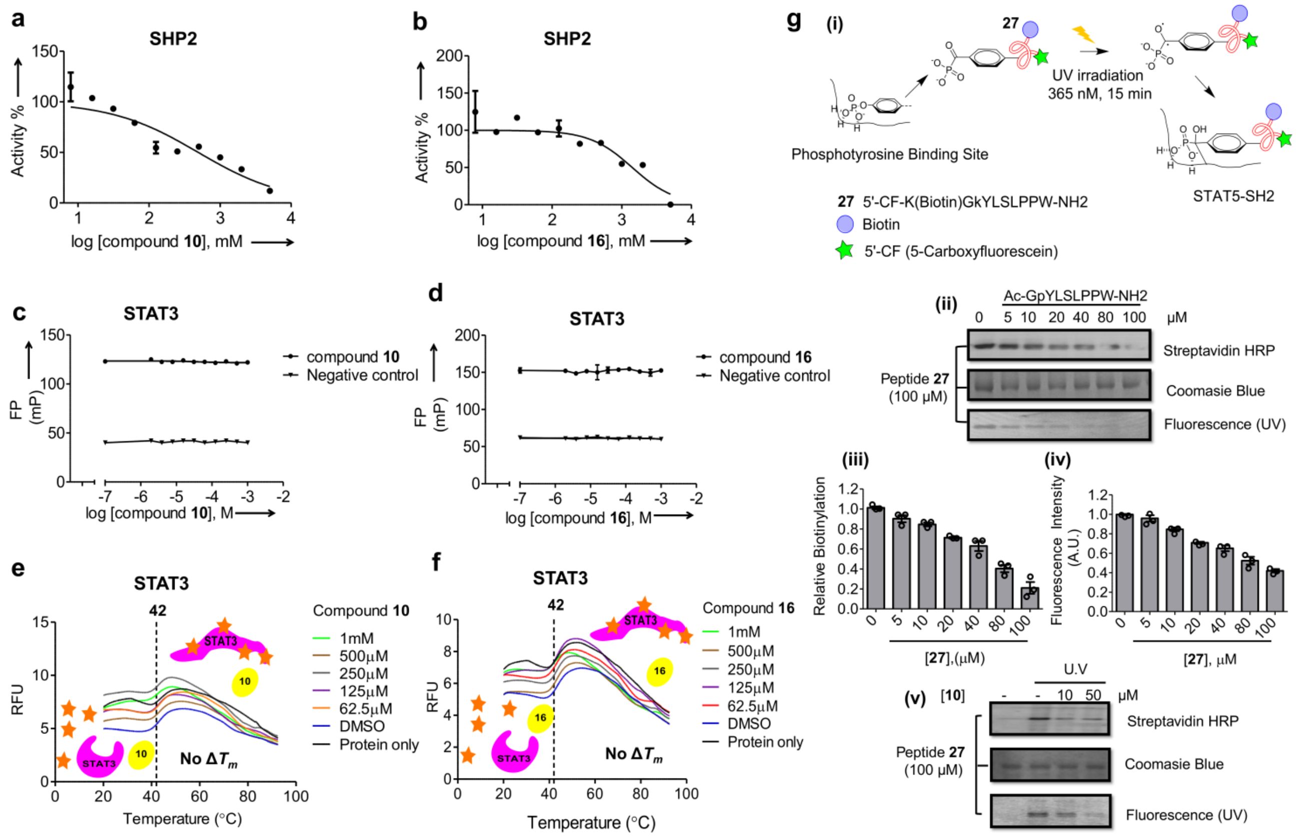

Supplementary Figure 4. Selectivity of compounds 10 and 16 for STAT5. a-b, Enzyme assays of compounds **10** and **16** with SHP2 protein (*n*=3). Both compounds showed no significant inhibition of the enzymatic activities of SHP2 protein at <1 mM. Dose-response fluorescence polarization curve illustrates no binding of compound **10** (**c**) and **16** (**d**) to recombinant STAT3 protein (*n*=3). Compound **10** (**e**) and **16** (**f**) did not induce thermal stability even at high concentration in STAT3 protein indicating binding specificity of both compounds towards STAT5 protein (data shown are one representative of *n*=3). **g** (**i**), Schematic illustration of the dual-labeled STAT5-binding peptide, 5-CF-K(biotin)GpcFLSLPPW-NH_2 **27** (CF= carboxyfluorescein, pcF=phosphonocarboxy-phenylalanine) photo-crosslink STAT5b upon UV irradiation. (**ii**) Photo-crosslinking of peptide **27** to STAT5 protein was displaced by the non-fluorescent; phosphotyrosines containing control peptide, 5-Ac-GpYLSLPPW-NH_2 affecting fluorescence intensity and biotinylation in a concentration dependent manner. (**iii-iv**) Relative STAT5 biotinylation and fluorescence intensity level were plotted using GraphPad Prism 5 after quantification using Image J software to reflect the efficiency of photo-crosslinking of peptide **27** (*n*=3).(**iv**) Compound **10** was able to interfere the photo-crosslinking event of peptide **27** indicating specific interaction of **10** with STAT5. Error bars denote mean ± S.D. (*n*=3). For an uncropped image of panels **g (ii)** and **g (v)** see Supplementary Figure **10**.

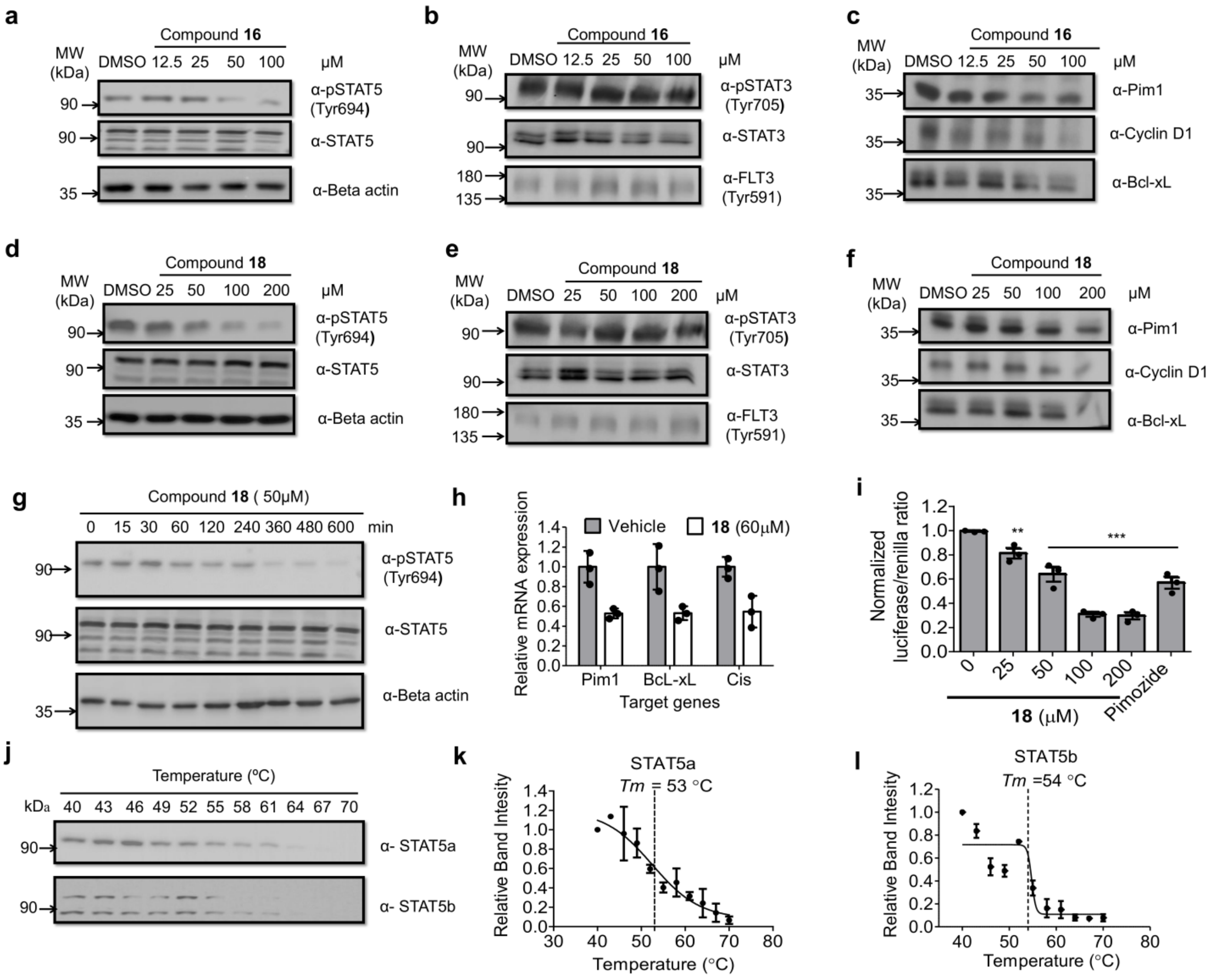

Supplementary Figure 5. Activity of compounds 16 and 18 in BaF3/FLT3-ITD cells. a,**d**, Phosphorylation of STAT5 in was reduced dose-dependent with compounds **16** and **18** with no effect on the endogenous STAT5 expression (*n*=3). **b**, **e**, Compounds **16** and **18** did not affect the endogenous expression and phosphorylation of STAT3 (*n*=3). **c**, **f**, Compounds BaF3/FLT3-ITD **16**, **18** impaired the expression of downstream target genes of STAT5 (*n*=3). **g**, Compound **18** reduced STAT5 phosphorylation steadily in a time-dependent manner (*n*=3). **h**, Expression of downstream targets of STAT5 was reduced after 18 h of treatment with compound **18** as quantified by quantitative PCR (*n*=3). **i**, Compound **18** inhibited transcriptional activity of STAT5 in BaF3/FLT3-ITD cells as measured by normalized Fluc/Rluc ratio in dual luciferase reporter assay (*n*=3). **j**, Representative Western-blot signals corresponding to STAT5a and b show a decrease in intensity at elevated temperatures (*n*=3). CETSA-melting curve of STAT5a (**k**) and b (**l**). Band intensities obtained from Western blot-analysis were related to the highest Western blot signal which has been set to 100%. Relative band intensities were plotted against incubation temperatures and fitted to Boltzmann sigmoidal curve. Error bars denote mean ± S.D. (*n*=3). For uncropped images of panels **a**, **b**, **c**, **d**, **e**, **f**, **g** and **j** see Supplementary Figure **10**.

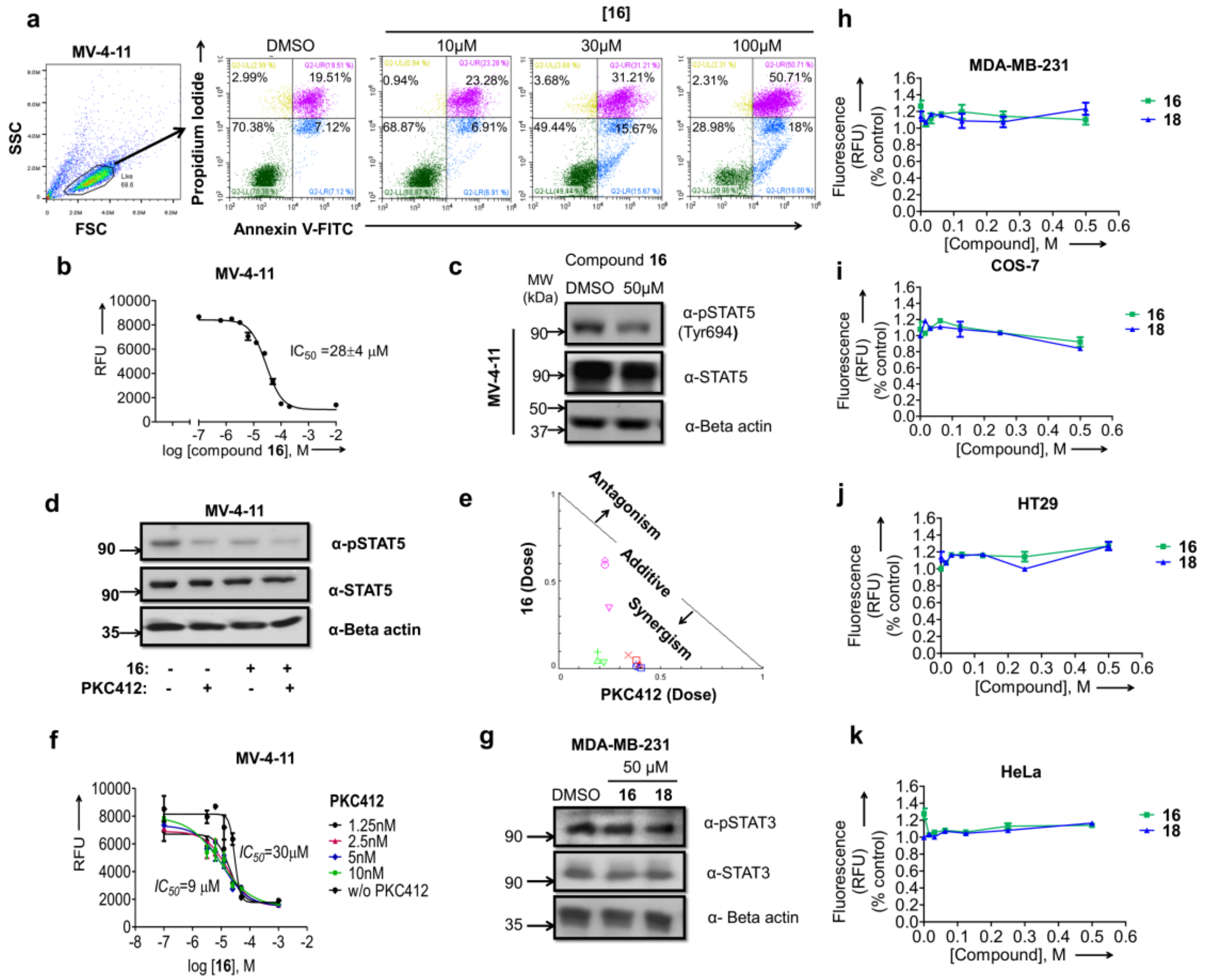

Supplementary Figure 6. Activity of compounds 16 on other human cell lines which are either STAT5 dependent (a-f) or not (g-k). a, Human myelomonoblastic cell line MV4-11, which carries the FLT 3-ITD mutation, was treated with **16** followed by annexin V/propidium iodide staining and flow cytometry (*n*=3). Debris were excluded using a forward scatter area (FSC-A) versus side scatter area (SSC-A) gate. **b**, Compound **16** inhibits the proliferation of MV-4-11 cells after 48 h as determined by the Alamar Blue assays (*n*=3). **c**, Western blot analysis of pSTAT5 inhibition in MV-4-11 cells after 6 h treatment with **16**. **d**, MV-4-11 cells were treated with **16** or PKC412 alone or in combination and incubated for 6 h and immunobloted with pSTAT5 to study synergistic effect of both compounds on STAT5 phosphorylation reduction (*n*=3). **e**, Analysis of the synergistic effect of the combination of **16** with PKC412. The calculated EC_{90} values for the combination were plotted as the fractional concentration (F_c) of **16** and PKC412 on the x and y axes (*n*=3). **f**, Effect of drug combination (**16** and PKC412) on cell viability as shown by Alamar blue assays (*n*=3). **g**, Compounds **16** and **18** show no effect on pSTAT3 in MDA-MB-231 cells, which rely heavily on pSTAT3 but not pSTAT5 activity for cell proliferation (*n*=3). **h-k**, Compounds **16** and **18** show no cytotoxicity in STAT5-independent cancer cell lines MDA-MB-231, COS-7, HT29 and HeLa. Error bars denote mean ± S.D. (*n*=3). For uncropped images of panels **c**, **d** and **g** see Supplementary Figure **10**.

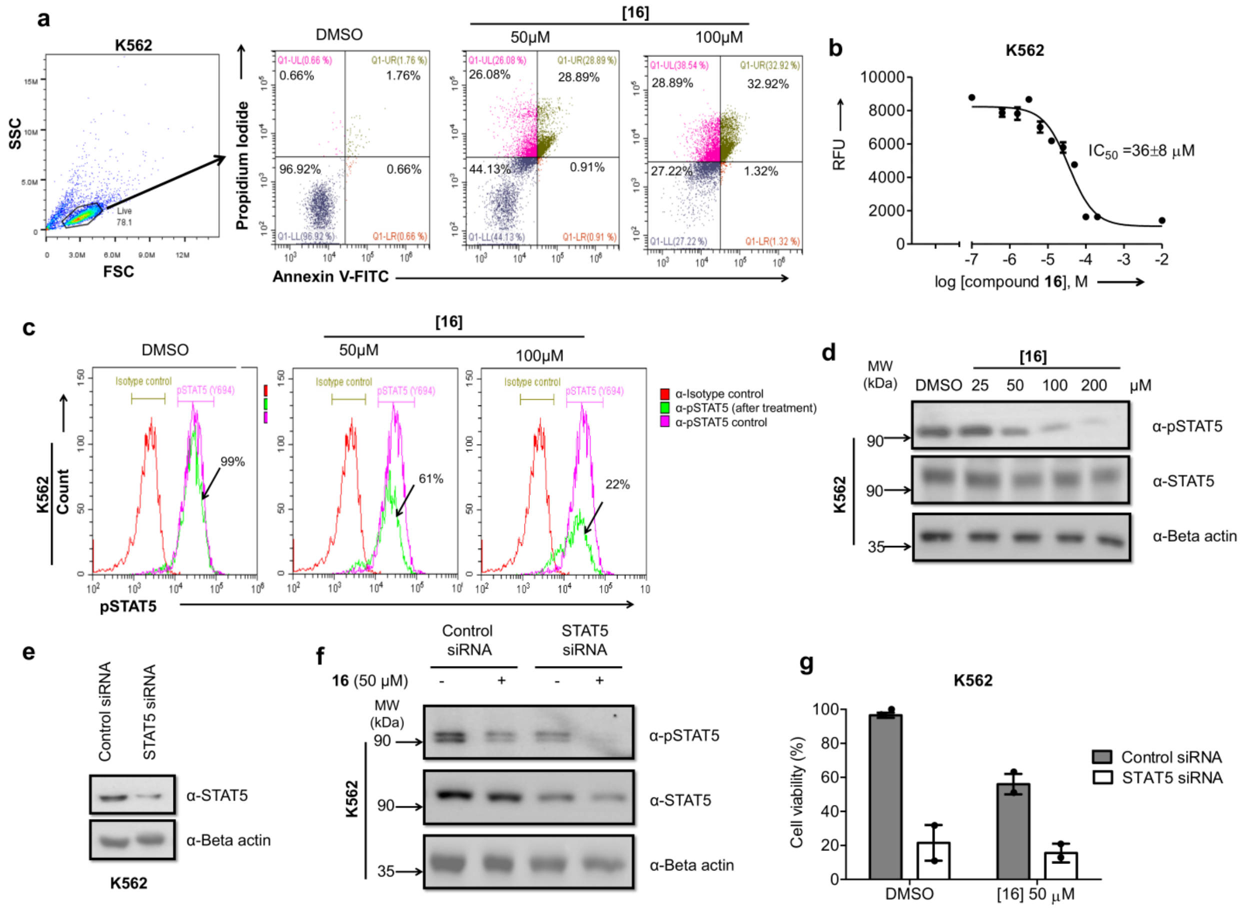

Supplementary Figure 7. Effects of STAT5 inhibitor 16 in K562 cells. a, Cells treated with compound **16** were stained with annexin V/propidium iodide and analyzed by flow cytometry (*n*=3). Debris was excluded using a forward scatter area (FSC-A) versus side scatter area (SSC-A) gate. **b**, Compound **16** inhibited the proliferation of K562 cells after 48 h as determined by the Alamar Blue assay (*n*=3). **c**, Intracellular levels of phosphorylated STAT5 (pSTAT5) were evaluated by flow cytometry after 6 h exposure of cells to compound **16** at a serial concentration (gating strategy as in Supplementary Fig. 11b, data shown are one representative of *n* = 3). **d**, Compound **16** blocked tyrosine phosphorylation of STAT5 in a dose dependent manner in K562 cells after 6 h treatment. Immunoblotting for beta-actin was used as a control for uniform protein loading (*n*=3). **e**, K562 cells were transfected with control siRNA or siRNA directed against STAT5 as indicated. Protein knockdown was confirmed by western blotting using antibodies against STAT5. β-Actin served as loading control (*n*=3). **f**, Western blot analyses of STAT5 protein in K562 cells treated with compound **16** and/or STAT5 siRNA (*n*=3). **g**, The effect of compound **16** on K562 cell viability was tested after genetic knockdown of STAT5. Cells were first transfected with STAT5 siRNA or control siRNA and incubated for 24 h before treated with compound **16** (50 μM) or DMSO for 48 h. Viable cells was distinguished using an ATP-dependent bioluminescence assay. Error bars denote mean ± S.D. (*n*=3). For uncropped images of panels **d**, **e** and **f** see Supplementary Figure **10**.

```
MuSTAT5a (aa589) WNDGAILGFV NKQQAHDLLI NKPDGTFLLR FSDSEIGGIT IAWKDFSPDR
HuSTAT5b (aa589) .......... .......... .......... .......... .......QE.

MuSTAT5a (aa639) NLWNLKPFTT RDFSIRSLAD RLGDLNYLIY VFPDRPKDEV FA
HuSTAT5b (aa639) MF...M.... .......... .......... .......... YS
```

Supplementary Figure 8. Alignment of SH2-domains mouse STAT5a (MuStat5a) to human STAT5b (HuSTAT5b)

a

```
>40310924.seq - ID: Klon40_5-GATC-Stat5B_intern1-2623426 on 2018/7/17-
6:17:24 automatically edited with PhredPhrap, start with base no.: 10
Internal Params: Windowsize: 20, Goodqual: 19, Badqual: 10,
Minseqlength: 50, nbadelimit: 1
TannGCCaGATGGGACCTTCCTCCTGAGATTCAGTGACTCAGAAATTGGCGGCATCACCATTGCTTGGAAG
                                 A
TTTGATTCTCAGGAAAGAATGTTTTGGGCTCTGATGCCTTTTACCACCAGAGACTTCTCCATTCGGTCCCT
                           642
AGCCGACCGCTTGGGAGACTTGAATTACCTTATCTACGTGTTTCCTGATCGGCCAAAAGATGAAGTATACT

CCAAATACTACACACCAGTTCCCTGCGAGTCTGCTACTGCTAAAGCTGTTGATGGATACGTGAAGCCACAG

ACCCCGGCGCGCCGATCCAGATCTCATCACCATCACCATCACTAAGCTTGGCACTGGCCGTCGTTTTACAA

CGTCGTGACTGGGAAAACCCTGGCGTTACCCAACTTAATCGCCTTGCAGCACATCCCCCTTTCGCCAGCTG

GCGTAATAGCGAAGAGGCCCGCACCGATCGCCCTTCCCAACAGTTGCGCAGCCTGAATGGCGAATGGCAGC

TTGGCTGTTTTGGCGGATGAGATAAGATTTTCAGCCTGATACAGATTAAATCAGAACGCAGAAGCGGTCTG

ATAAAACAGAATTTGCCTGGCGGCAGTAGCGCGGTGGTCCCACCTGACCCCATGCCGAACTCAGAAGTGAA

ACGCCGTAGCGCCGATGGTAGTGTGGGGTCTCCCCATGCGAGAGTAGGGAACTGCCAGGCATCAAATAAAA

CGAAAGGCTCAGTCGAAAGACTGGGCCTTTCGTTTTATCTGTTGTTTGTCGGTGAACGCTCTCCTGAGTAG

GACAAATCCGCCGGGAGCGGATTTGAACGTTGCGAAGCAACGGCCCGGAGGGTGGCGGGCAGGACGCCCgc

cAtAacggCcaGgCATC
```

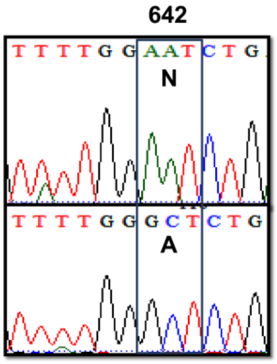

b **c**

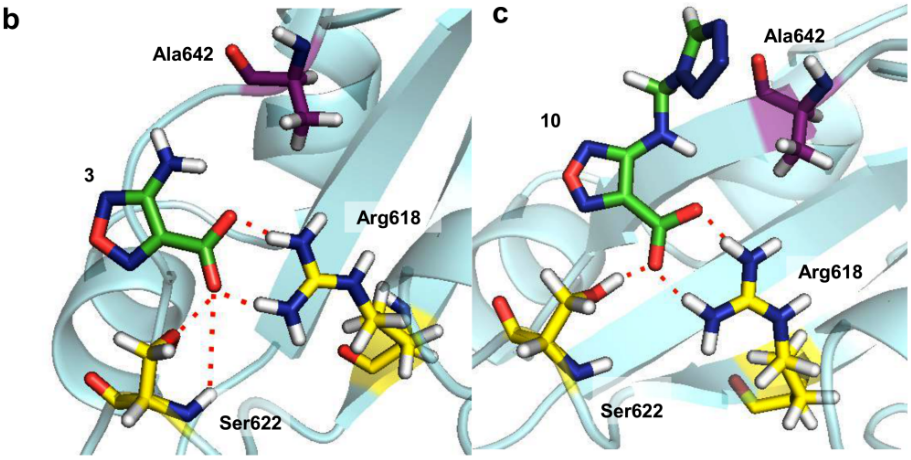

Supplementary Figure 9. Site-directed mutagenesis N642A on the STAT5b sequence. **a**, Sanger sequencing data confirmed that the AAT codon (Asn) is replaced to GCT (Ala) at the amino acid site 642. Molecular illustration of key binding residues, Asn642 substituted with alanine and affected the ligand binding for compound **3** (**b**) and **10** (**c**) by losing essential H-bonds. STAT5 protein was shown as cyan ribbon; key interacting residues were depicted as yellow stick; Residue subjected to alanine mutation was shown as purple sticks; hydrogen bonds with key residues in the hydrophilic binding pocket of the STAT5-SH2 domain were illustrated as red dashed lines.

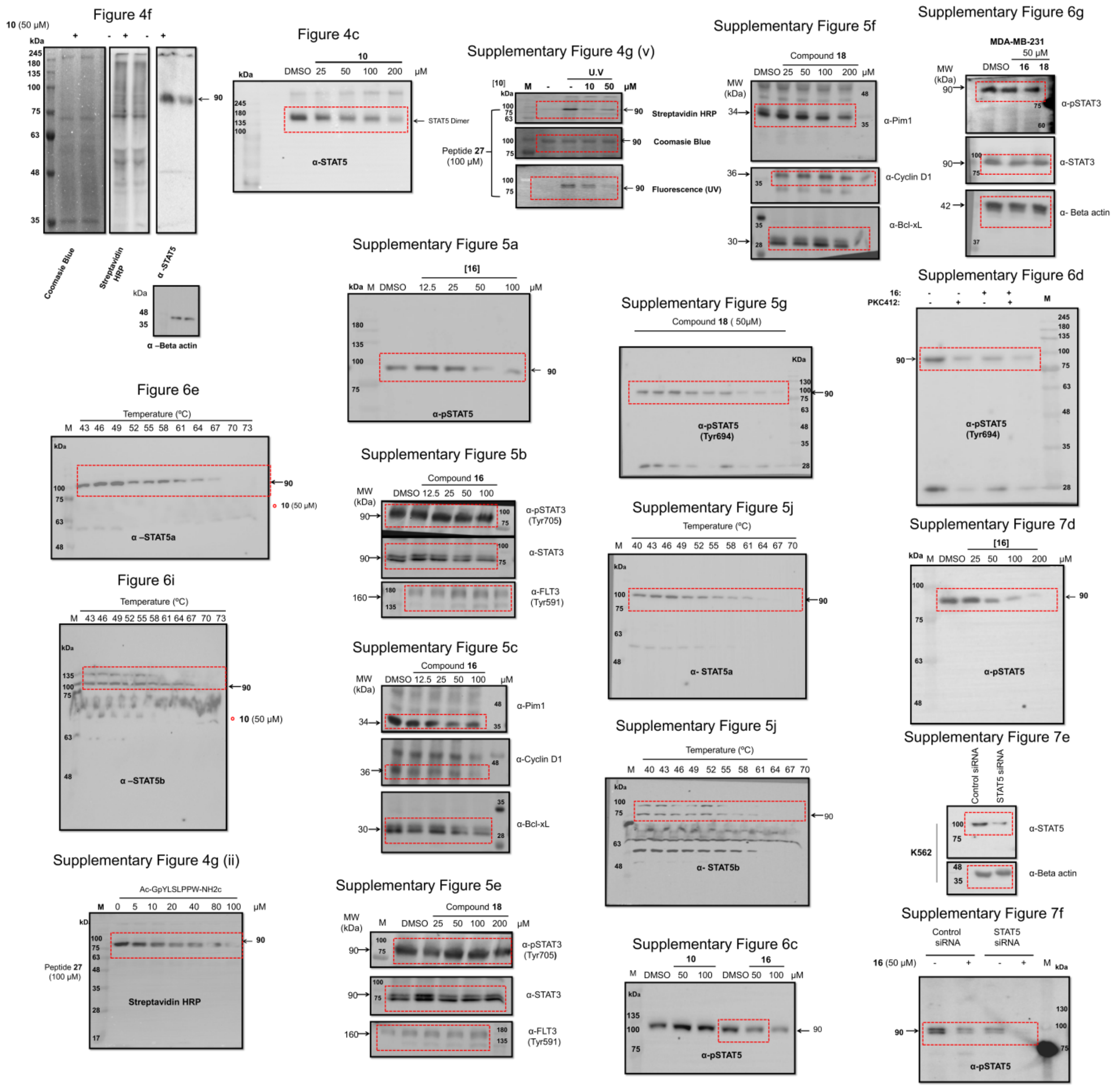

Supplementary Figure 10. Uncropped pictures of Western blots from the corresponding cropped Western blots shown in Figures 4 and 6 and Supplementary Figures 4, 5, 6, and 7. Molecular weight markers are indicated. Figure subpanels are indicated for each respective blot.

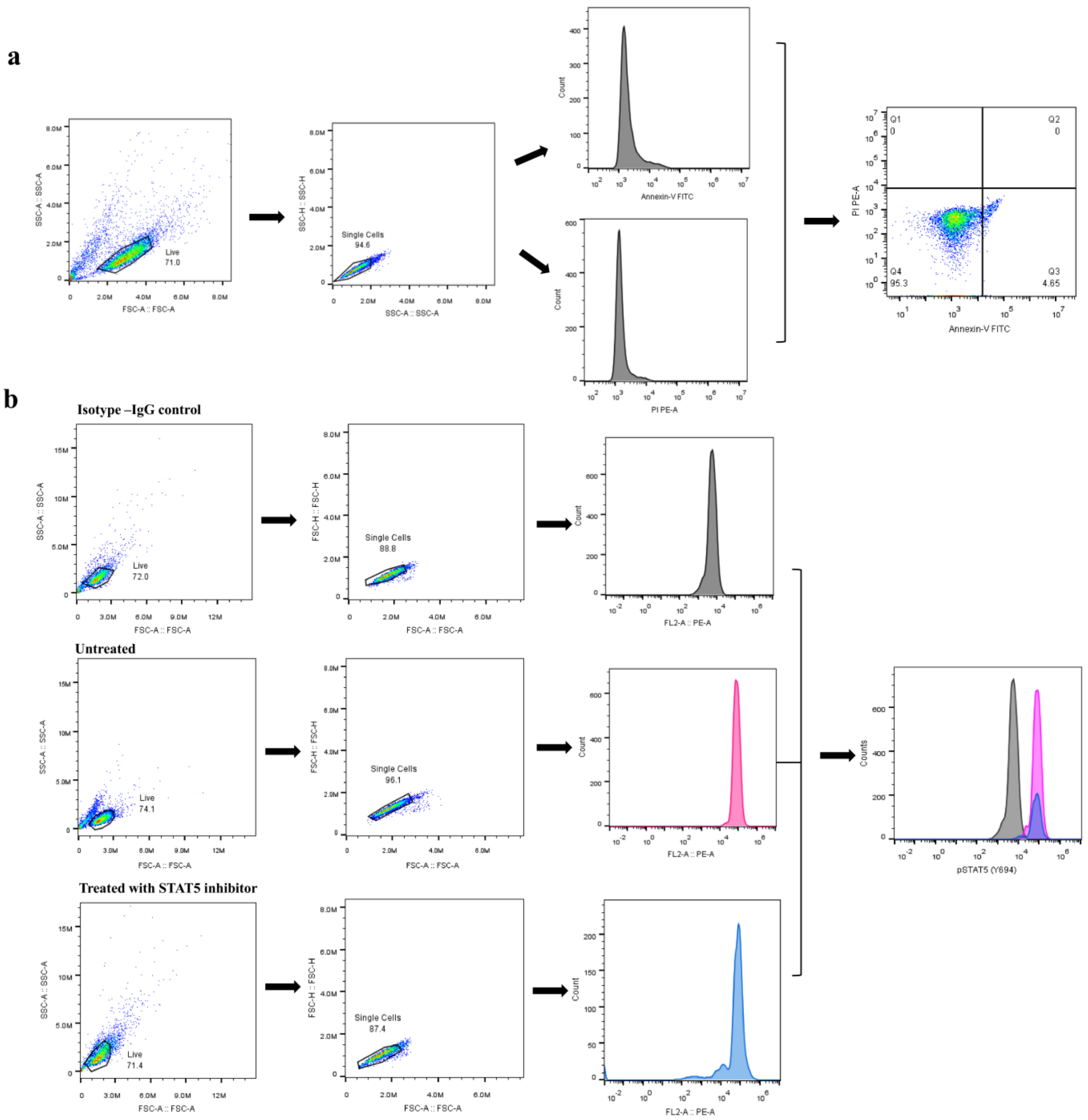

Supplementary Figure 11. Gating strategies used for flow cytometry stainings. a, Gating strategy used for flow cytometry staining presented in Figures 5e (i), 6a, and 7a to determine apoptosis using fluorescein isothiocyanate (FITC)-conjugated annexin-V and dual stained with phycoerythrin (PE)-conjugated propidium iodide to exclude necrotic cells. Debris were excluded using a forward scatter area (FSC-A) versus side scatter area (SSC-A) gate. Single cells (singlets) were then selected on a FSC-A versus FSC-W plot to exclude signaling data from doublets. **b**, Phospho-flow cytometry gating strategy used for the analysis of STAT5 phosphorylation presented in Figure 5e (ii) and Supplementary Figure 7c using PE Mouse Anti-Stat5 (pY694) and PE conjugated rat anti-mouse IgG monoclonal antibody as isotype control.

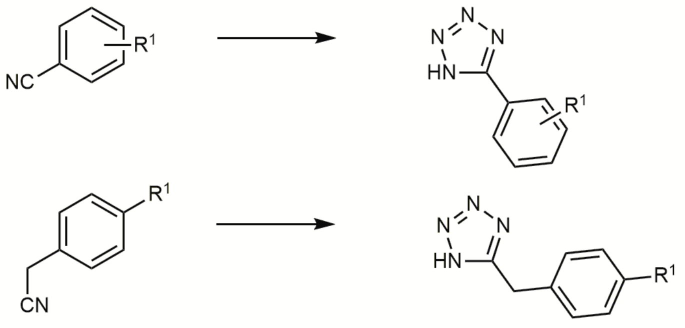

Supplementary Figure 12. Reaction scheme for the synthesis of tetrazole derivatives

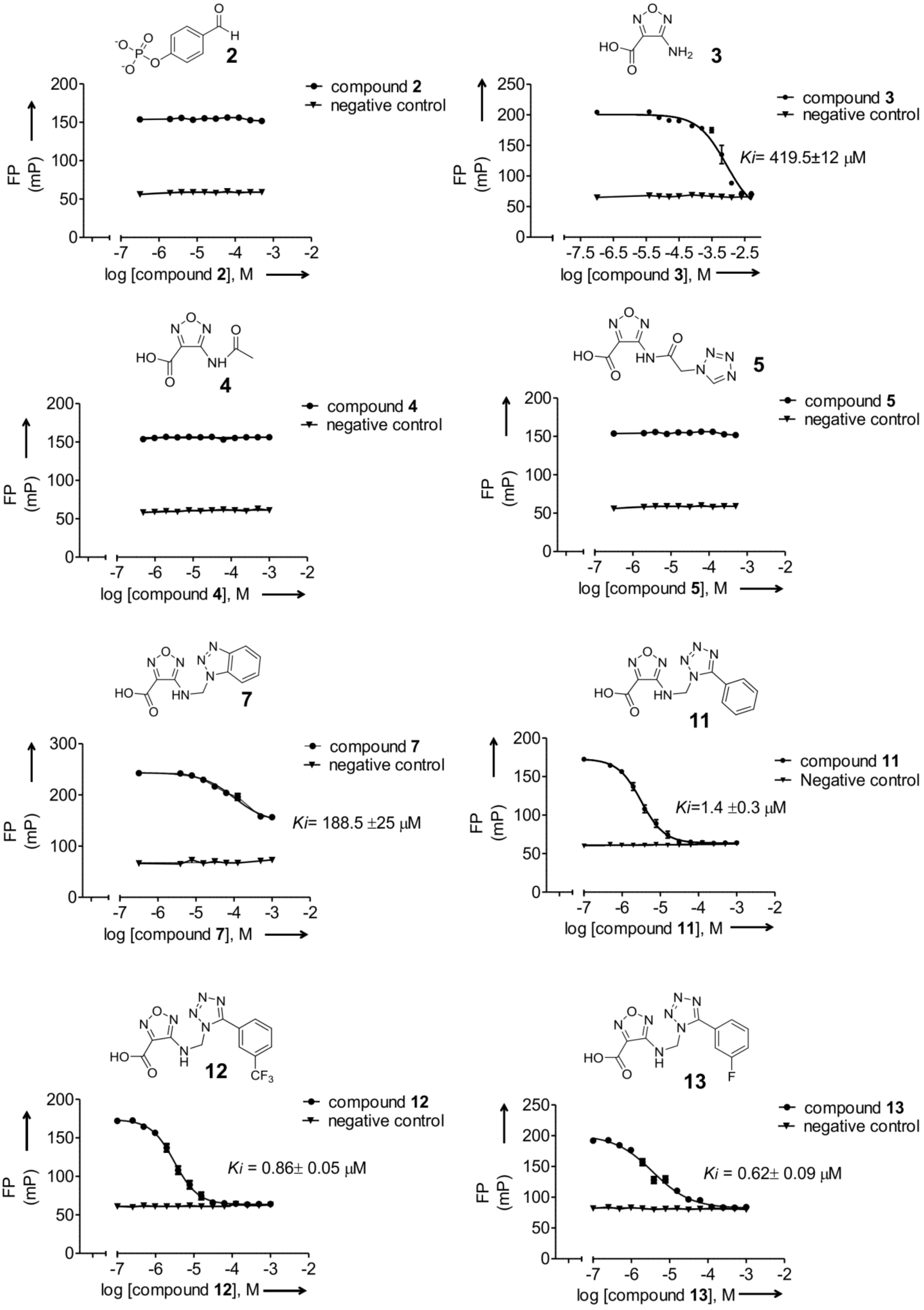

Supplementary Figure 13. Fluorescence polarization binding assays of STAT5 inhibitors. Dose-response fluorescence polarization (FP) curves for the competitive binding of compounds **2-13** to recombinant STAT5-SH2 domain as in Supplementary Table 1. Error bars denote mean ± S.D. (*n*=3).

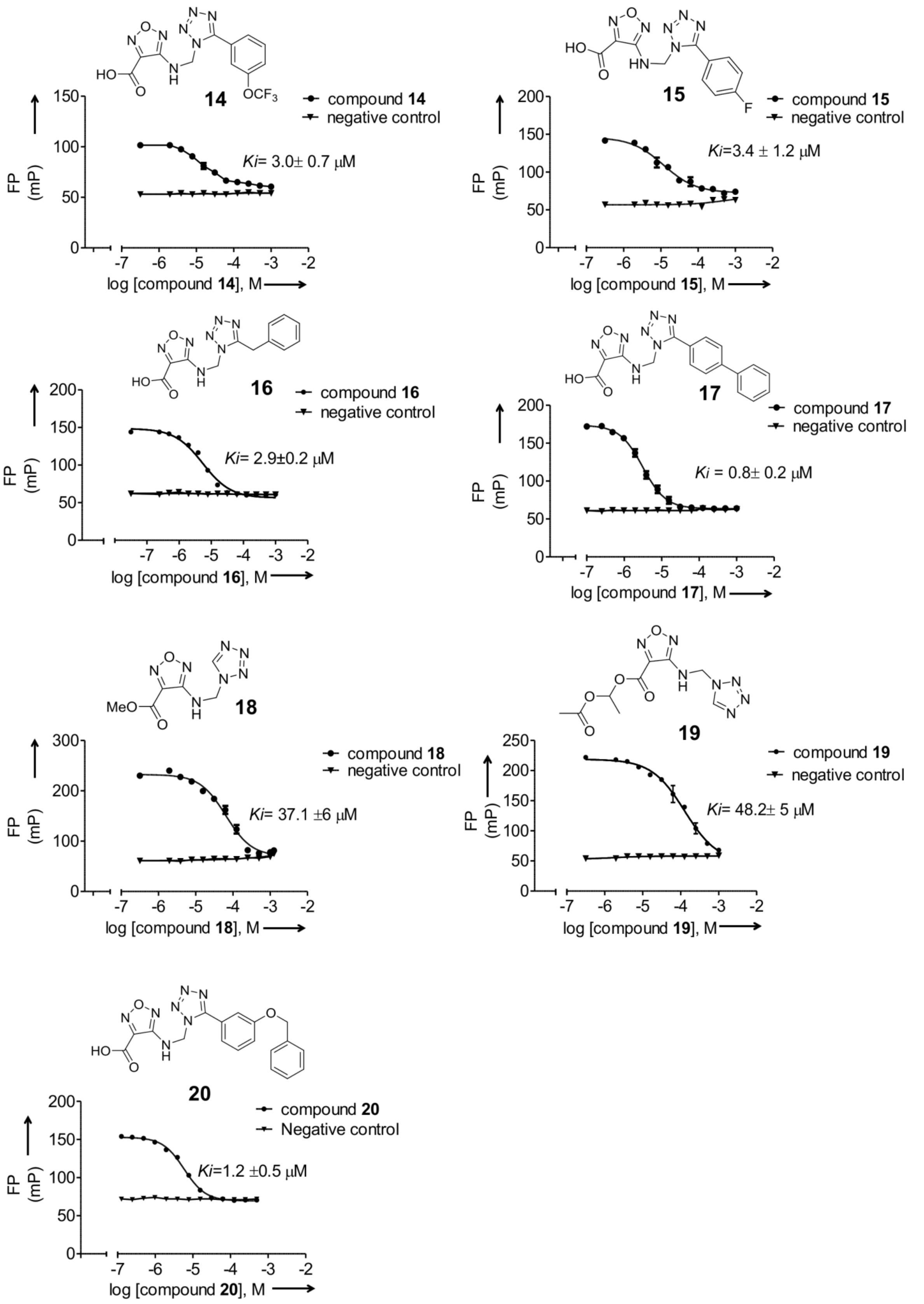

Supplementary Figure 14. Dose-response fluorescence polarization (FP) curves for the competitive binding of compounds **14-20** to recombinant STAT5-SH2 domain as in Supplementary Table 1. Error bars denote mean ± S.D. (*n*=3).

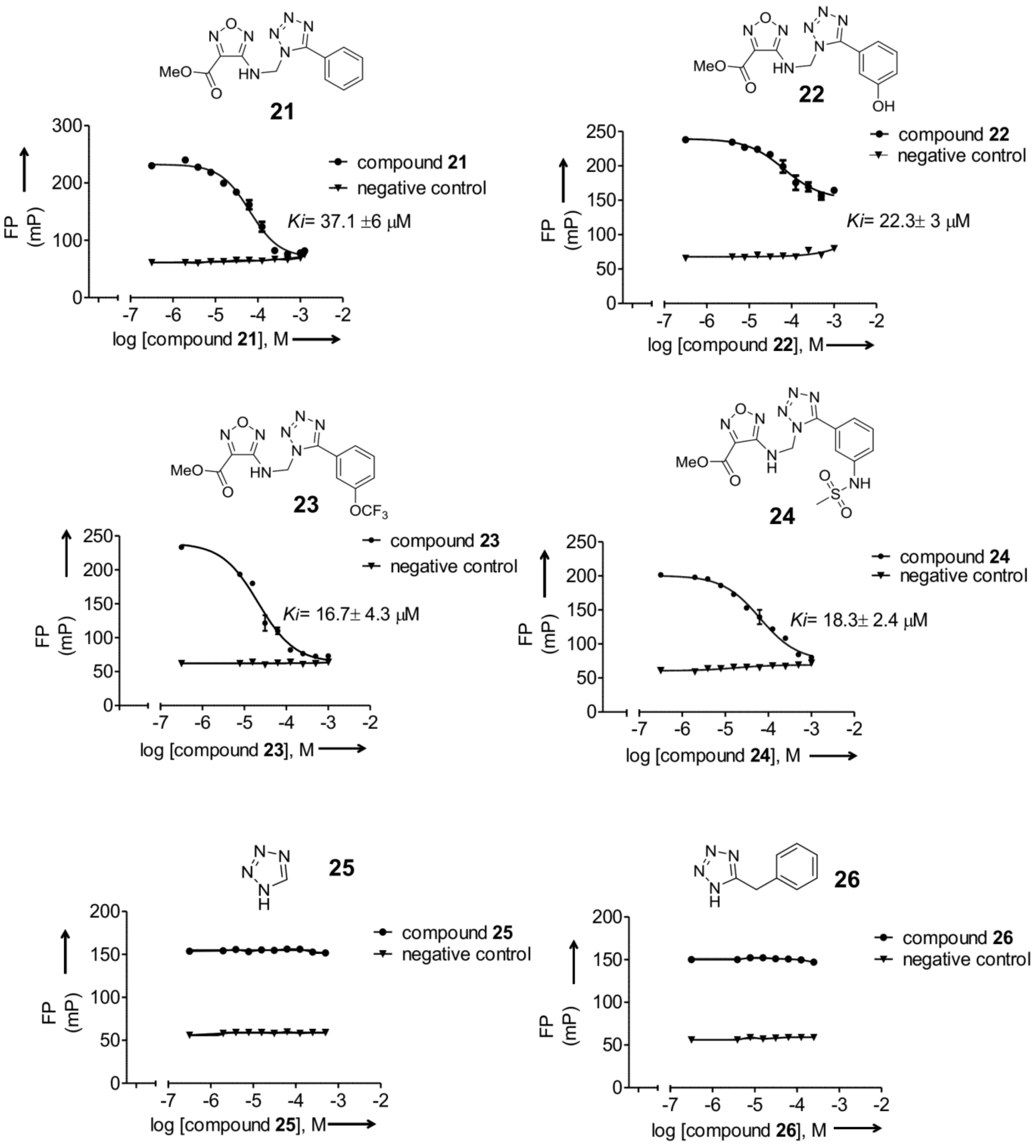

Supplementary Figure 15. Dose-response fluorescence polarization (FP) curves for the competitive binding of compounds **21**-**26** to recombinant STAT5-SH2 domain as in Supplementary Table 2. Error bars denote mean ± S.D. (*n*=3).

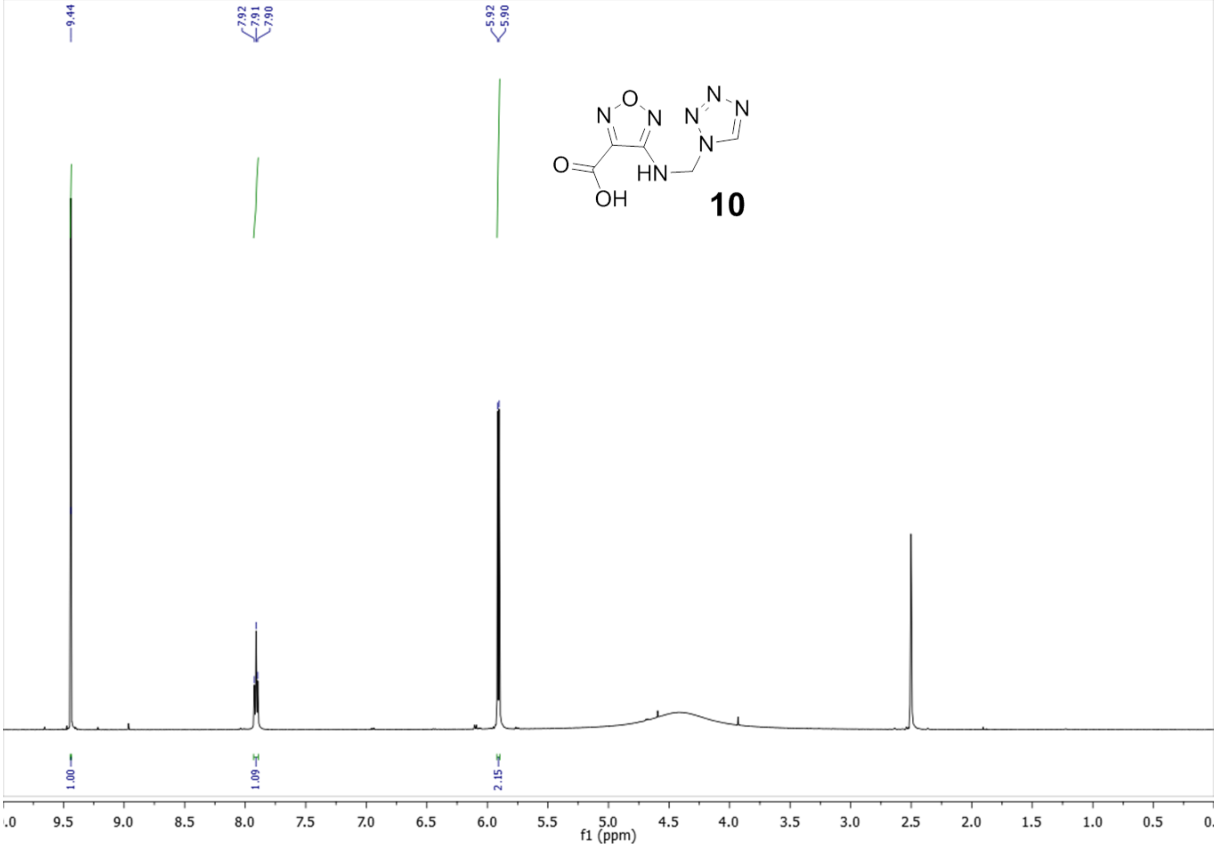

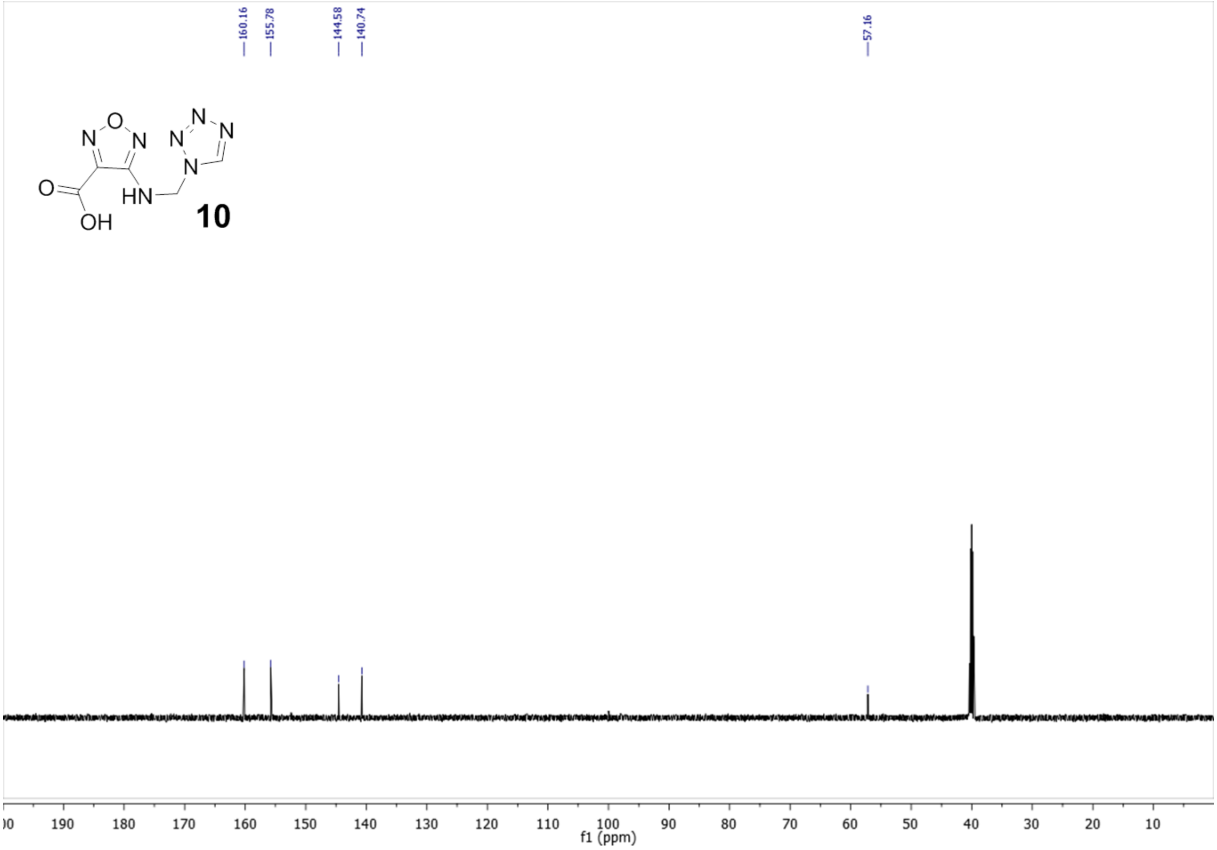

Supplementary Figure 16. ^{1}H- and ^{13}C-NMR spectrum of **10**

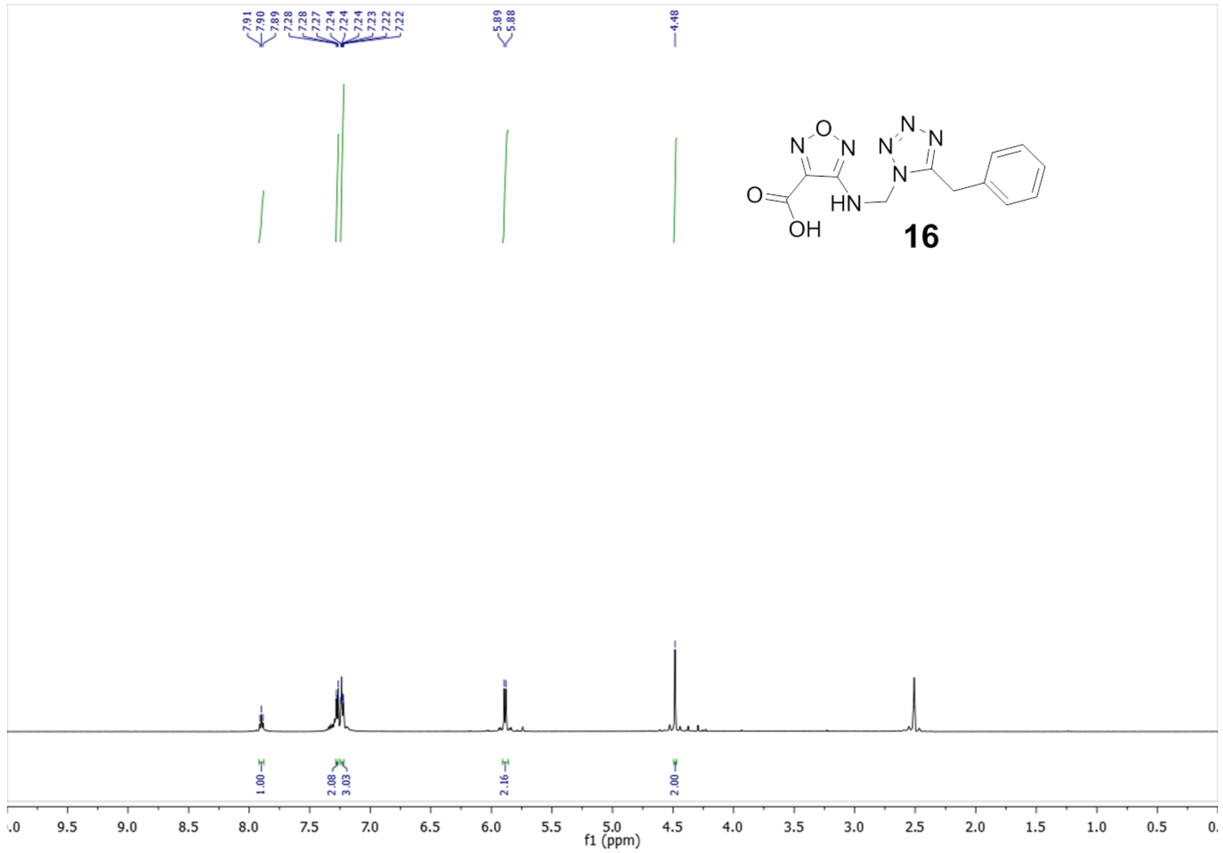

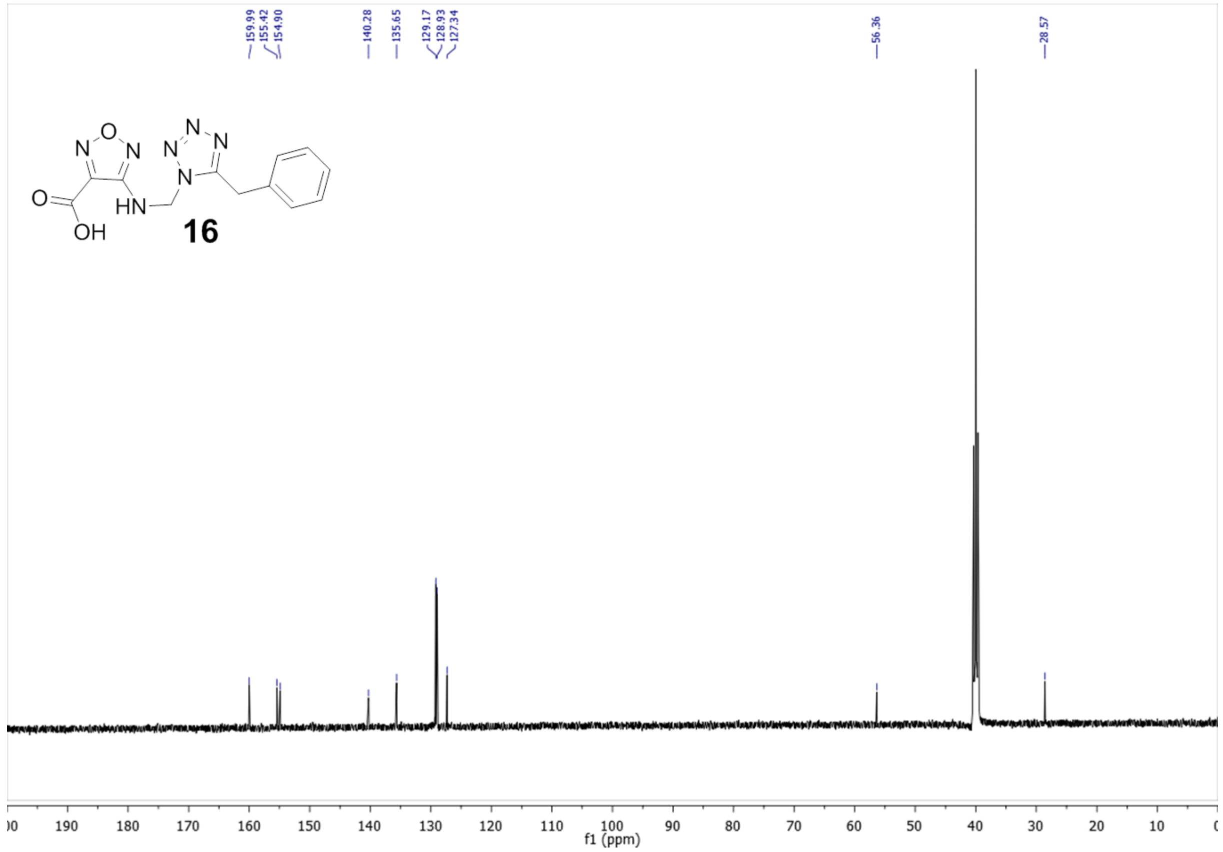

Supplementary Figure 17. ^{1}H- and ^{13}C-NMR spectrum of **16**

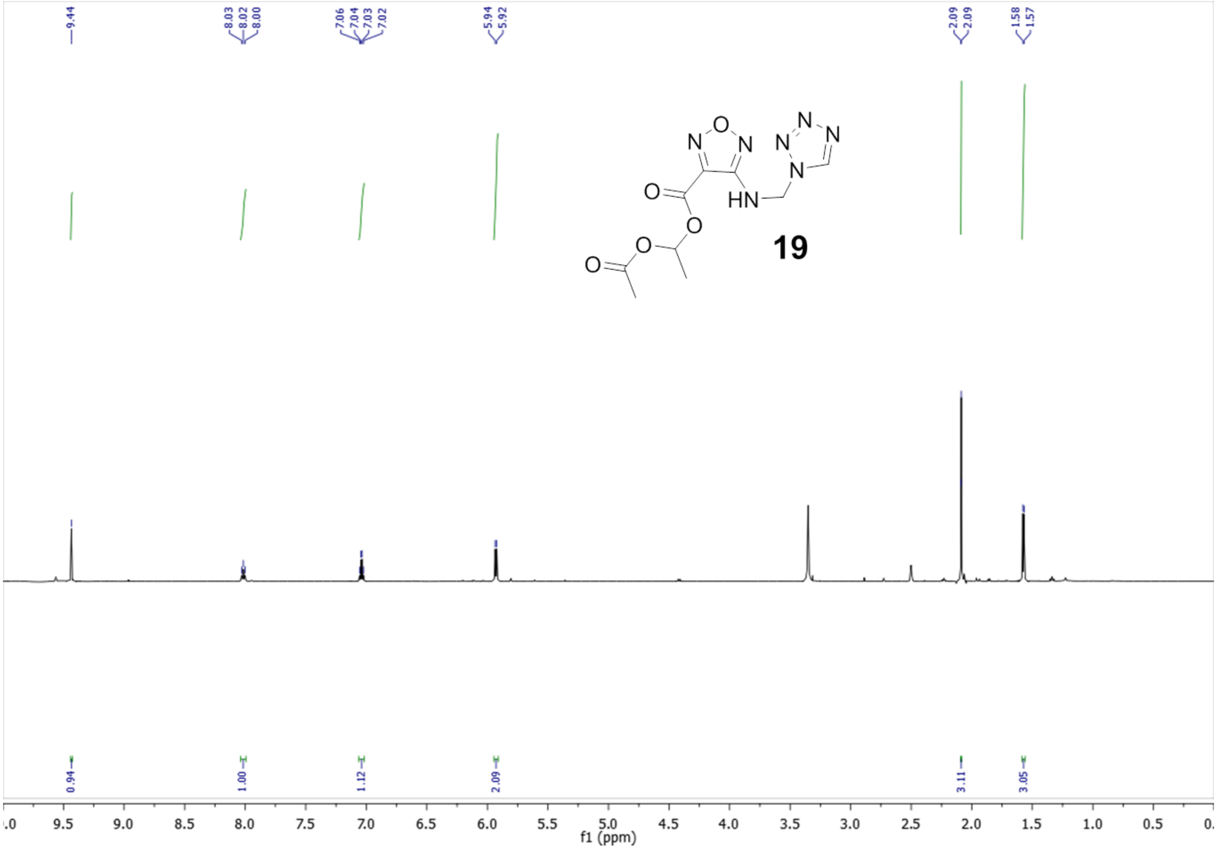

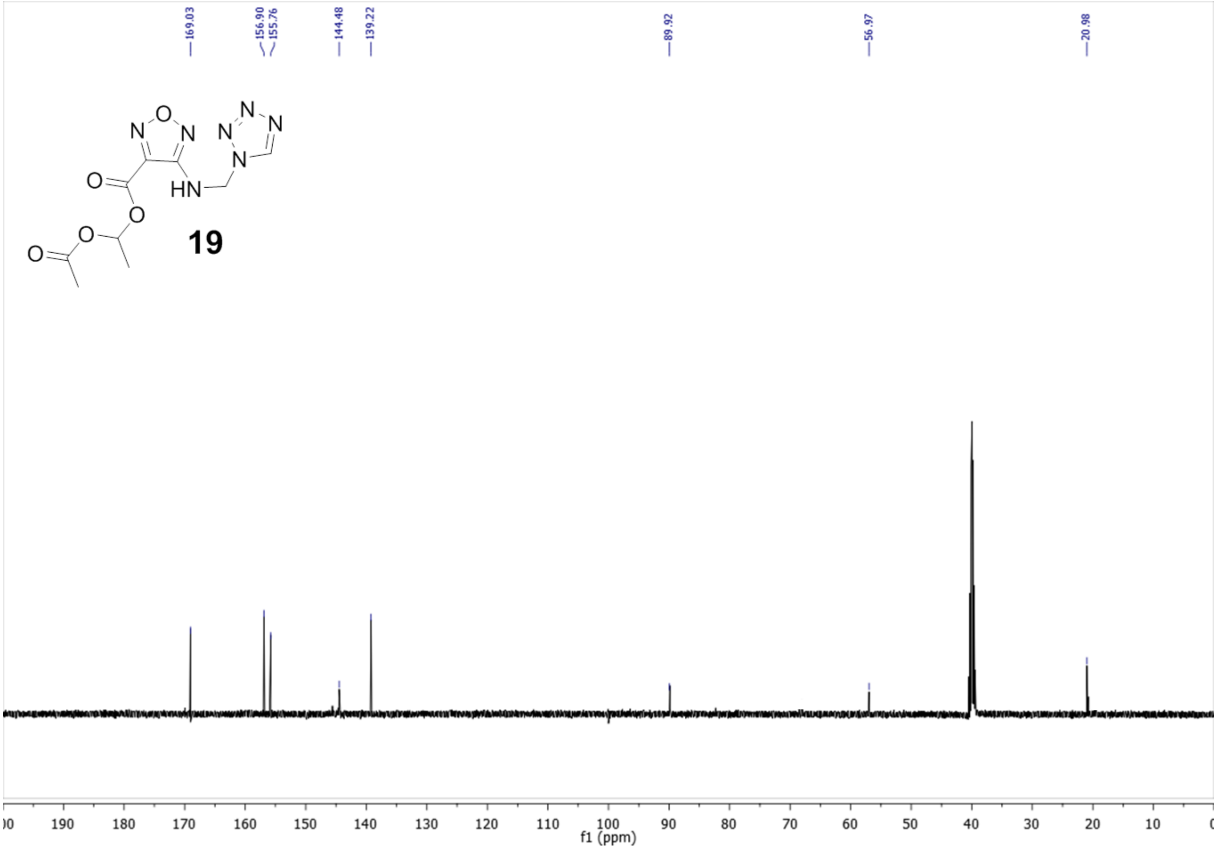

Supplementary Figure 18. ^{1}H- and ^{13}C-NMR spectrum of **19**

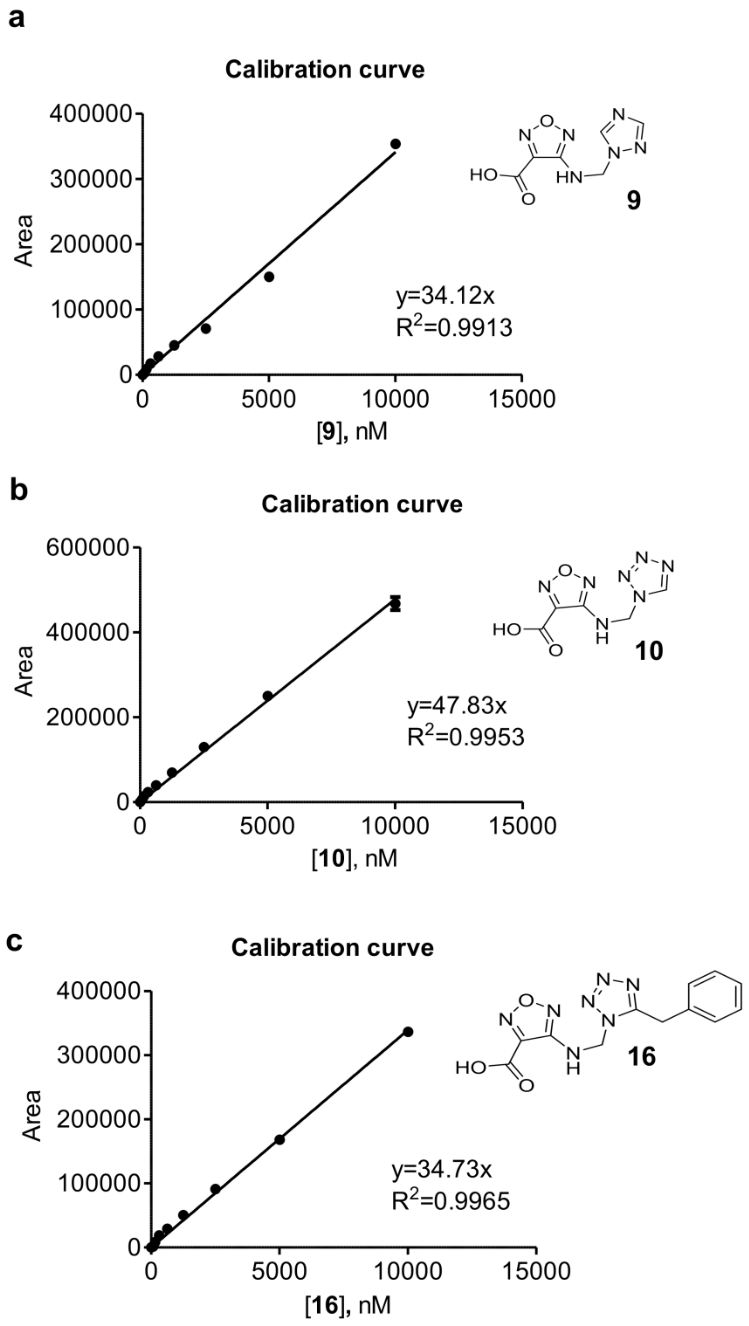

Supplementary Figure 19. a-c Standard calibration curves of 6 different concentrations of compound **9**, **10** and **16**. Error bars denote mean ± S.D. (n=3).

Supplementary Tables

Supplementary Table 1. STAT5 inhibitors and their respective K_I values and ligand efficiencies.

Cpd #	Structure	K_I (µM)	LE (kJmol^{-1} NA^{-1})
**1**	5-CF-GY*LSLPPW-NH_2	0.055±0.006	0.42
**2**	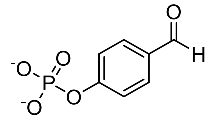	>2500	n.a.
**3**	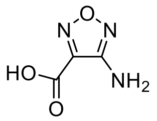	419.5±12	2.14
**4**	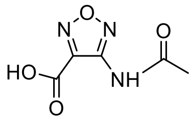	>2500	n.a.
**5**	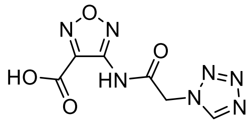	> 2500	n.a.
**6**	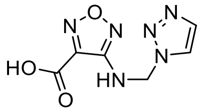	190.6±24	1.41
**7**	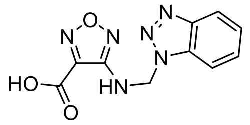	188.5±25	1.12
**8**	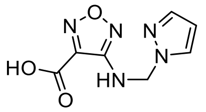	121.5±25	1.49
**9**	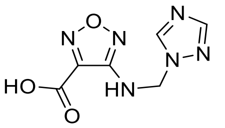	47.5±8.5	1.64
**10**	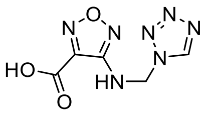	1.4±0.5	2.23
**11**	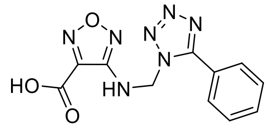	1.4±0.3	1.59
**12**	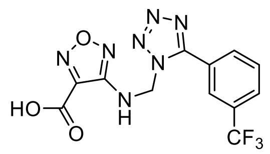	0.9±0.05	1.38
**13**	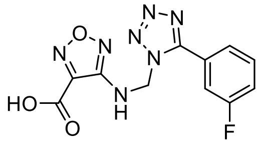	0.6±0.09	1.38
**14**	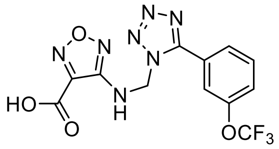	3.0±0.7	1.21
**15**	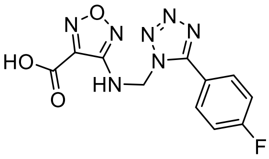	3.4±1.2	1.42
**16**	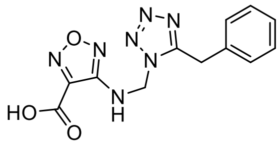	2.9±0.2	1.44
**17**	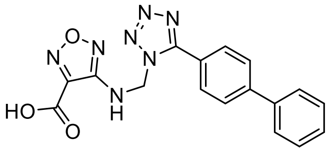	0.8±0.2	1.30
**18**	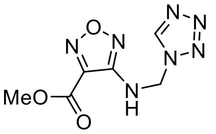	37.4±1.2	1.58
**19**	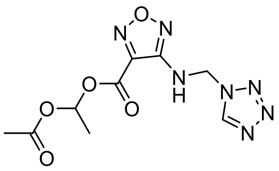	4.6±1.7	1.45
**20**	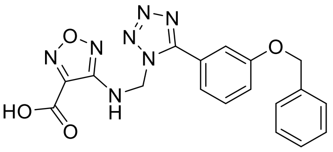	1.2±0.5	1.16

Conversion of IC_{50} values into K_I values was carried out as described[1] and ligand efficiency was calculated using the equation derived.[2]

n.a. = not applicable, NA = number of non-hydrogen

Supplementary Table 2. Ester derivatives (**21-24**) of STAT5 inhibitors**.**

Cpd #	Structure	K_I (μM)	LE ($kJmol^{-1}$ NA^{-1})
**21**	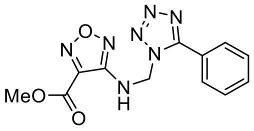	37.1±6	1.15
**22**	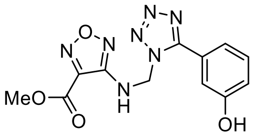	22.3±3	1.21
**23**	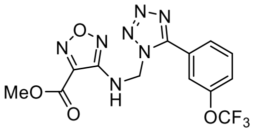	16.7± 4.3	1.01
**24**	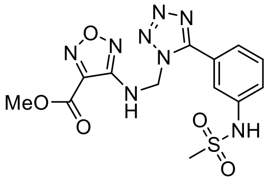	18.3±2.4	1.00
**25**	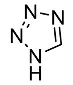	>2500	n.a.
**26**	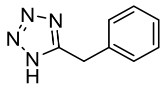	>2500	n.a.

n.a. = not applicable; NA = number of non-hydrogen atoms.

Supplementary Table 3. List of primers used in this study.

Genes	Forward Primer	Reverse Primer
Pim-1(m)	TCTTCTGGCAGGTGCTG	GGTAGCGAATCCACTCTG
Bcl-xL(m)	ATGGCAGCAGTGAAGCAAGC	ACGATGCGACCCCAGTTTACTC
Cis(m)	CTGGACTCTAACTGCTTGTC	TAGGCAGCACCGAGTCAC
S9(m)	GGGATGTTCACCACCTG	GCAAGATGAAGCTGGATTAC

Supplementary Methods

Expression and purification of STAT5b and STAT5b-N642A

Expression of the truncated version of STAT5b (aa 137-747) cloned into a modified pQE70, with N-terminal MBP-tag and C-terminal His-tag was conducted on autoinduction medium (overnight express / Novagen, cat. 71491, Merck, Germany). Cells were grown to an optical density (O.D.) of 0.3 at 37 °C, then the temperature was reduced to 20 °C for further 48 h of expression. Comparable soluble expression levels were obtained with Rosetta2 (DE3) (cat.71400, Novagen, Merck, Germany) and BL21 (DE3) pLysS (cat.69451, Novagen, Merck, Germany). The protein was purified by Ni-chelating chromatography followed by gel filtration (Superdex 200 / 10 mM HEPES pH 7.8, 100 mM NaCl, 1 mM EDTA, 1 mM DTT, 10% glycerol).A yield of 15 mg MBP-STAT5b-His per liter of culture was obtained and aliquots (200 µl of 3.2 mg ml^{-1}) were quick-frozen and stored at –80 °C, ready for use[3]. Site-directed mutagenesis was performed using PCR-based site directed mutagenesis with high fidelity PWO DNA polymerase (cat.11644947001, Roche, Germany) and confirmed by Sanger sequencing (Supplementary Figure 9). The mutant proteins were expressed and purified as described above. Purified mutant proteins were further concentrated by ultrafiltration (Amicon 30-kDa MW cut-off; cat. UFC903024, Milipore Corporation, Billerica, MA).

Thermal shift assays

Thermal shift assays[4] were performed with fragments **3**, **25**, and **26** in 96-well PCR plates (cat HSL9901 Bio-Rad Laboratories, Richmond, CA). Assays were carried out using 500 nM of MBP-STAT5b protein mixed with serial concentration of fragments in the presence of 20x Sypro Orange (cat.S6650, Thermo Scientific, Braunschweig, Germany). Assay buffer contains 300 mM HEPES, pH 7.4, 175 mM NaCl and 1% DMSO. The PCR plates were

sealed with optical seal, shaken for 15 min, and centrifuged. Thermal scanning (20 to 95°C at 1°C/min) was performed using a real-time PCR setup (Light Cycler, Roche Diagnostics, Mannheim, Germany) and fluorescence intensity was measured after every 0.3 s. Curve fitting, melting temperature calculation and report generation on the raw data were performed using GraphPad Prism 5 software.

Fluorescence polarization assays and screening

Ca. 17000 compounds and fragments from the ChemBioNet library were tested in a fluorescence polarization (FP) assay to investigate their ability to bind to STAT5b-SH2 domain by displacing the fluorophore-labeled peptide 5-carboxyfluorescein-GY (PO_3H_2) LSLPPW-NH_2 **1**. Purified compounds were tested in the same assay. The peptide purity was >95% and the assays were performed at room temperature. The final concentration of buffer components used was 10 mM HEPES (pH 7.5), 1 mM EDTA, 0.1% Igepal CA-630, 50 mM NaCl, and 5% DMSO and the final concentration of protein used was at 125 nM. The protein was first added to the black 384-well plate (Corning cat.CLS3676, Sigma Aldrich, Taufkirchen, Germany) followed by test compounds and fluorophore-labeled peptide. The plates were centrifuged, and measured using Safire^2 well plate reader (Tecan, Crailsheim, Germany) after 15 min incubation at room temperature. For testing secondary site binding of primary hit fragments, the same assay was conducted in the presence of 4-formylphenyl phosphate **2** as described earlier. For specificity analysis, FP assay was conducted with 100 nM GST-tagged, full length human STAT3 protein (cat. S54-54G-50, SignalChem, Richmond, BC, Canada) and 10 nM fluorophore-labeled peptides (5-carboxyfluorescein-GY (PO_3H_2) LPQTV-NH_2). The assay buffer contains 50 mM NaCl, 10 mM HEPES (pH 7.5), 1 mM EDTA, 0.01% Triton-X100 and 2 mM dithiothreitol). The test compounds were serially diluted and incubated with STAT3 protein at room temperature for 1 h followed by 10 nM of fluorophore-labeled peptide[3,5]. The mixture was centrifuged and incubated for 30 min at room

temperature before FP was recorded using the MTP reader. For analysis of the data GraphPad Prism 5 was used. Ligand efficiencies (LE) were calculated using the equation LE = $-\Delta G^{o}/HA$ with ΔG^{o} being the standard free energy of binding in kJM^{-1} and HA the number of heavy, non-hydrogen atoms[6].

Homology modeling of STAT5b

Template was adopted from the C-terminal region (Trp589 to Ser680, 92 amino acids) of crystal structure, 1Y1U[7][http://dx.doi.org/10.2210/pdb1Y1U/pdb]. The alignment was taken from Lin et al.[8] Seven side chains were mutated to turn the SH2-domain of mouse STAT5a to human STAT5b using Sybyl8.1.[9] The side chain conformation of Arg618 from the template structure of 1Y1U was manually adjusted so that it may interact in a bidentate coordination with the acidic group of the substrate like observed in other SH2 domains (e.g. 1BKM [http://dx.doi.org/10.2210/pdb1BKM/pdb][10] or 1O46 [http://dx.doi.org/10.2210/pdb1O46/pdb][11]).

Preparation of STAT5 conformations

The software AutoDockTools[12] was used to convert homology modeled STAT5 and ligands to PDBQT from the PDB files. Polar hydrogens were assigned and Gasteiger charges were added and finally structures were saved in the PDBQT file format for docking.

AutoDock Vina docking

The *AutoGrid* and *AutoDock* Vina[13] procedures were used to conduct the grid point energy calculations of the receptor and the binding pose scoring of the ligands, respectively. The binding conformations of the ligands were optimized using the Lamarckian Genetic Algorithm (LGA), where the initial population size for each ligand was set to 2,500,000 and the grid was set

at 30x 30x 30 Å, centered around the phosphotyrosines binding site in the SH2 domain of STAT5. The exhaustiveness was set to 100 for a better global minimum search.

BINding ANAlyzer

Docking conformation with minimum $E_{FreeBind}$ was loaded into BINANA[14] for descriptors calculations. BINANA is a python implemented algorithm that assist in characterizing binding of inhibitor-receptor complex. Receptor and ligand files were prepared by MGLTools 1.5.6[12] in PDBQT format. BINANA descriptors consist of (i) close contacts, (ii) electrostatic interactions; (iii) hydrophobic contacts, (iv) hydrogen bonds, (v) salt bridges and (vi) π interactions.

Detection of ligand 16 formation via mass spectrometry

Extracted ion chromatography was performed with reaction mixtures containing 250 nM of MBP-STAT5b protein, 250 µM (IC_{20}) 4-amino-furazane-3-carboxylic acid **3**, equimolar amount (250 µM) of one heteraryl nucleophile and FA with a total volume of 100 µl. The reaction mixtures were vortexed to mix thoroughly and incubated overnight at room temperature and was analyzed using a HPLC/QTOF-MS instrument by Agilent, consisting of an Infinity 1290 UHPLC coupled to a 6550 iFunnel QTOF. After 12 h each sample was analyzed in triplicate by injecting (10 µl) into the LC/MS instrument and the ligation products were identified by their molecular weights and by comparison of the retention times of synthetic reference. Calibration curves for hit compounds **9**, **10**, and **16** are given in Supplementary Figure 19. Eluents were mixtures of water and acetonitrile (with 0.1% formic acid). Injection volume was set to 10 µL. Samples were eluted using gradient elution of started off with 97:3 (water/acetonitrile) for 1 min followed by 95:5 to 5:95 over 5 min. Flow rate was set to 0.3 ml min^{-1}. The QTOF is equipped with an electrospray ionization-source used with the following parameters: negative ion mode, fragmentor voltage 175 V, capillary

voltage 4000 V, nozzle voltage 1000 V, gas temperature 200 ºC, gas flow 14 l min^{-1}, stealth gas temperature 350 ºC, stealth gas flow 11 1 min^{-1}. The reference masses 121.050873 mz^{-1} and 922.009798 mz^{-1} were used for reference ion correction. The mass range was set to 100-1000 and a scan rate of 1 spectrum/s was chosen. Due to the low complexity of sample matrix, the instrument was run in full-scan mode (ms-only) with sufficient selectivity and sensitivity. In order to have a clear separation and lower interference from the buffer salts, HPLC-flow from 1.3 to 5 min was directed to the mass detector. Data processing and integration were performed using the MassHunter software by Agilent Technologies with mass window set to 10 ppm. All control experiments were run consecutively. Experiments were carried out as describe below for data collection and analysis in Supplementary Figure 3.

Lane 1: Blank reaction, negative control

For a negative control, 4-amino-furazane-3-carboxylic acid **3** (250 µM, IC_{20}), equimolar amounts (250 µM) of a hetaryl nucleophile, e.g. 5-benzyl-1H-tetrazol **26**, and formaldehyde were incubated for 24 h at room temperature in MOPS buffer (50 mM) at pH 7.4 in the absence of the protein template (MBP-STAT5b-SH2). The reaction mixture was analyzed using the method described above.

Lane 2: Protein-induced ligation

4-Amino-furazane-3-carboxylic acid **3** (250 µM, IC_{20}) and equimolar amounts (250 µM) of a hetaryl nucleophile, e.g. 5-benzyl-1H-tetrazol **26** or and formaldehyde were incubated for 24h at room temperature in MOPS buffer (50 mM) at pH 7.4 in the presence of the protein template (MBP-STAT5b-SH2). The reaction mixture was analyzed using the method described above.

Lane 3 and **4**: **Protein-induced reaction in the presence of high affinity FP probe 1**

Ligations were carried out after pre-incubating MBP-STAT5b-SH2 in MOPS buffer (50 mM) at pH 7.4 with 100 nM and 300 nM of **1**, respectively, for 1 h at room temperature followed by the addition of 4-amino-furazane-3-carboxylic acid **3** (250 µM), an equimolar amount (250

μM) of a 1H-tetrazole, and of FA in total assay volume of 100 μl. The reaction mixture was incubated for 24 h at room temperature and was analyzed using method as described above.

Lane 5 and **6: Protein-induced ligation in the presence of competitive inhibitor 10**

Ligations were carried out by pre-incubating MBP-STAT5b (final concentration 250 nM) with 1 and 5 μM of **10** for 1 h at room temperature followed by addition of 250 μM (IC_{20}) 4-amino-furazane-3-carboxylic acid **3**, equimolar amounts (250 μM) of one hetaryl nucleophile and FA. The reaction mixtures were incubated for 24 h at room temperature and was analyzed using method as described above.

Lane 7: Protein-induced Mannich ligation experiments with maltose binding protein (MBP) in place of MBP-STAT5-SH2 protein

Ligations were carried out by mixing 1 μM (in excess) of MBP protein, 250 μM (IC_{20}) 4-amino-furazane-3-carboxylic acid **3**, equimolar amount (250 μM) of one heteraryl nucleophile and FA The reaction mixtures were incubated for 24 h at room temperature and was analyzed using method as described below.

Lane 8: In-situ reaction at pH 5.0, positive control

Mannich ligations were carried out by mixing 4-amino-furazane-3-carboxylic acid **3** (250 μM, IC_{20}), an equimolar amount (250 μM) of one heteraryl nucleophile and formaldehyde yielding a total assay volume of 100 μl in 50 mM MOPS buffer, pH 5.0. The reaction mixtures were incubated overnight at room temperature and was analyzed using method as described above.

Activity measurement of SHP-2 using a DiFMUP assay

An enzyme assay using 6,8-difluoro-4-methylumbelliferyl phosphate (DiFMUP)[15] as a substrate was employed for the determination of SHP-2 activity. Test compounds were dissolved in dimethyl sulfoxide (DMSO) at a concentration of 100 mM and the assay was carried out at a final DMSO concentration < 1 %. The phosphatase reactions were performed at room temperature in 384-well black plate, clear flat bottom, low flange, non-binding

surface (Corning, cat.3766, Sigma Aldrich, Taufkirchen, Germany) using a final volume of 20 µl and the DiFMUP assay buffer contained a final concentration of 50 mM MOPS (pH = 6.5), 200 mM NaCl, 0.03 % Tween-20, 1 mM DTT (freshly added prior to each measurement) and 2.5 nM SHP-2 (final concentration). SHP-2 and tested compound in buffer solution were incubated for 30 min at r.t. The reaction was started by adding DiFMUP (Invitrogen, cat. D6567, 10 µM, correspond to the experimentally determined K_M values of the enzymes) and the measurements were performed on a microplate reader (infinite M1000 Pro, Tecan) using excitation and emission wavelengths of 360 nm and 460 nm. Measurements were performed in triplicate and the IC_{50} values were calculated with GraphPad Prism 5. IC_{50} values were converted into the corresponding K_I values applying the Cheng Prusoff equation $K_I = IC_{50} / (1 + [S]/K_M)$[16].

Photo-crosslinking and competitive displacement of probe 27

Peptide probe **27** (100 µM) was incubated with 200 µL of recombinant MBP-STAT5 SH2 protein in the binding buffer (50 mM HEPES, pH 7.5, 200 mM NaCl, 2 mM $MgCl_2$, 0.1% tween-20, 20% glycerol, 2 mM PMSF, Roche Complete EDTA-free protease inhibitor cocktail) for 1 h at 4 °C. The samples were then irradiated at 365 nM using a UV transilluminator for 15 min at 4 °C. For competitive displacement studies, non fluorescent; phosphotyrosines containing control peptide (0-100 µM) was added together with peptide probe **27** and incubated with recombinant MBP-STAT5 SH2 protein for 1 h at 4 °C prior to UV photo-crosslinking for 15 min at 4 °C.[17] SDS sample buffer (final concentration 1x) were added to the buffer and boiled before running on SDS PAGE followed by western blotting.

Preparation for nuclear extracts and cytoplasmic extracts

Nuclear extracts and cytoplasmic extracts were prepared from BaF3/FLT3-ITD cells using the Nuclear Extraction kit (Active Motif, Carlsbad, CA, USA) according to the manufacturer's protocol.

Preparation of whole cell lysate

BaF3/FLT3-ITD cells were grown in suspension at 37 ºC in a humidified atmosphere with 5% CO_2 in RPMI medium containing 10% dialyzed FBS. After harvesting, cell pellets were washed twice with PBS and frozen with liquid N_2. To prepare whole cell lysates, the cell pellets were resuspended in a hypotonic buffer (10 mM HEPES, pH 7.5, 2 mM $MgCl_2$, 0.1% tween-20, 20% glycerol, 2 mM PMSF, and Roche Complete EDTA-free protease inhibitors) and incubated for 10 min at 4 ºC. The suspension was centrifuged at 16000 xg for 15 min at 4 ºC and the supernatant was kept for use later. The pellets were resuspended in a high-salt buffer (50 mM HEPES, pH 7.5, 420 mM NaCl, 2 mM $MgCl_2$, 0.1% tween-20, 20% glycerol, 2 mM PMSF, and Roche Complete EDTA-free protease inhibitors) and incubated for 30 min at 4 ºC . The suspension was then centrifuged at 16000 xg for 15 min at 4 ºC and the supernatant was combined with the soluble fraction in hypotonic buffer to give the whole cell lysates.

Cellular thermal shift assay (CETSA)

Cellular thermal shift[18] assays were performed to monitor the target engagement of **10** and **16** for STAT5a and STAT5b protein in BaF3/FLT3-ITD cells. Briefly, cell lysate from a total of 2×10^6 BaF3/FLT3-ITD cells was collected, diluted in PBS and separated in identical aliquots. Lysates were divided into 45 µl in each of PCR tubes and heated individually at different temperatures with Thermocycler (Biometra, Göttingen, Germany). The heated lysates were centrifuged and the supernatants were analyzed by SDS-PAGE followed by

immunoblotting analysis by probing with anti-STAT5a (C-6) sc271542 and STAT5b (G-2) sc-1656 (Santa Cruz Biotechnology) antibody, respectively.

Photo-crosslinking and competitive displacement of 27 with recombinant MBP-STAT5 SH2 protein

Peptide probe **27** (100 μM) was incubated with 200 μL of recombinant MBP-STAT5 SH2 protein in the binding buffer (50 mM HEPES, pH 7.5, 200 mM NaCl, 2 mM $MgCl_2$, 0.1% tween-20, 20% glycerol, 2 mM PMSF, Roche Complete EDTA-free protease inhibitor cocktail) for 1 h at 4 ºC. The samples were then irradiated at 365 nM using a UV transilluminator for 15 min at 4 ºC. For competitive displacement studies, non-fluorescent; phosphotyrosine-containing control peptide (0-100 μM) was added together with peptide probe **27** and incubated with recombinant MBP-STAT5 SH2 protein for 1 h at 4 ºC prior to UV photo-crosslinking for 15 min at 4 ºC. SDS sample buffer (final concentration 1x) were added to the buffer and boiled before running on SDS PAGE followed by western blotting.

Western blot analysis and immunoprecipitation

Cells were seeded at $0.5x10^6$ and allowed to grow overnight followed by 6 h incubation with test compounds (0.1% DMSO) before protein extraction with M-PER™ Mammalian Protein Extraction Reagent (Pierce) containing 1% (vol/vol) complete protease inhibitor cocktail (Roche Molecular Biochemicals) and 1% (vol/vol) phosphate inhibitor cocktail (Sigma). For Western blotting, 15 μg of protein from each sample were then separated on a 10% SDS-PAGE and transferred to a PVDF membrane. The blots were blocked with TBST buffer (20 mM Tris-HCl [pH 7.4], 140 mM sodium chloride, and 0.05% Tween 20) containing 5% BSA at room temperature for 1 h, washed 3 times in TBST buffer, and incubated with primary antibody overnight at 4°C. The membranes were then incubated with HRP-conjugated

secondary antibody at room temperature for 1 h. The reaction products were detected using Syngene Pxi4 imager and quantified by Image J[19].

STAT5 luciferase reporter assay

BaF3/FLT3-ITD cells (5x10^6 cells in 0.3 ml) were co-transfected with a ratio of 10:1 pGL4.52[luc2P/STAT5RE/Hygro] vector (cat.E4651, Promega, Germany) and pRL Renilla Luciferase Control Reporter vectors (cat. E2231, Promega, Germany) as a transfection efficiency control in Opti-MEM medium via electroporation. (ECM 830 electroporator, BTX Instruments, Holliston, MA). The transfected cells were then seeded in a 24-well plate and treated with serial dilution of compounds for 6 h. The cells were collected 48 h after transfection, and the luciferase activities in the cell lysates were determined using the dual luciferase reporter assay system (cat. E1910, Promega, Germany). Each transfection was performed in triplicate and repeated twice.

RNA isolation and real-time PCR

RNA was harvested using NucleoSpin RNA kit (cat. 740955 Macherey-Nagel, Germany). cDNA was generated using the SuperScript™ II Reverse Transcriptase (cat. 18064014, Thermo Scientific) and Real-Time quantitative RT-PCR was performed using Transcriptor High Fidelity cDNA Synthesis (cat. 5081955001, Sigma Aldrich, Germany) and Light-Cycler 480 SYBR Green I Master Kits (cat. 04707516001, Roche, Germany) on a Light-Cycler 480 Real-Time PCR System according to instructions given by the manufacturer (Roche, Germany). Data were evaluated using the Light-Cycler 480 software (1.5). Quantitative reverse transcription polymerase chain reaction (RT-PCR) was performed using primers as described[20]. Data are expressed as the arithmetic means of the relative change of RNA expression ± S.D. of 3 replicates and S9 expression was used as an internal control. The sequences of the oligo-primers used were tabulated in Supplementary Table 3.

Isothermal dose response fingerprint experiments (ITDRF)

BaF3/FLT3-ITD cells were grown in suspension at 37 ºC in a humidified atmosphere with 5% CO_2 in RPMI medium containing 10% dialyzed FBS. Approximately 2×10^6 cells were collected, washed with PBS buffer and replaced with fresh RPMI with 10% FBS. Cells were then separated in identical aliquots before treated with compound **16** at a final concentration between 0 and 100 µM (0.1% DMSO) and incubated under standard tissue culture conditions for 6 h. Cells were re-suspended in PBS supplemented with Complete EDTA-free protease inhibitors (Roche), aliquots (100 µl) containing equal cell numbers in PCR tubes were prepared. Tubes were incubated at approximately the T_m of the proteins of interest as determined by CETSA melting curve experiments, 60 °C (T_m for actin) for 3 min, followed by room temperature for 3 min. The tubes were centrifuged (300 g, 3 min, 4 °C). The supernatant was removed and cells suspended in lysis buffer (100 mM HEPES, 300 mM NaCl, 2% NP-40 and 10 mM EDTA, pH 7.4), supplemented with protease inhibitors. The tubes were incubated at 4 °C for 1 h, with vortexing every 20 min. Samples were centrifuged for 30 min at 16,000 x g at 4 °C to pellet cell debris and precipitated proteins[21]. The supernatants were analyzed by SDS-PAGE followed by immunoblotting analysis by probing with anti-STAT5a (C-6 cat. sc271542) and STAT5b (G-2, cat. sc-1656) (Santa Cruz Biotechnology) antibody, respectively.

Transient transfection with STAT5 siRNA

Transient transfection for knockdown of endogenous STAT5 proteins was prepared by using STAT5 siRNA (STAT5 siRNA (h), cat.sc-29495) from Santa Cruz Biotechnology (Santa Cruz, CA) and Control siRNA (control siRNA-A,cat. sc-37007) was used a negative control. Cell were transfected with STAT5 siRNA using siRNA transfection reagent (cat.sc-29528, Santa Cruz, CA) according to the manufacturer's instructions. Selective silencing of STAT5 was confirmed by western blot analysis. To assay the effect of STAT5 knockdown on cell

viability, cells were first transfected with STAT5 siRNA or control siRNA and incubated for 24 h before treated with compound **16** (50 μM) or DMSO for 48 h. Viable cells was distinguished using an ATP-dependent bioluminescence assay (cat. G7570, CellTiter-Glo, Promega).

Cell proliferation assay

The effect of compounds on cell lines was evaluated by In Vitro Toxicology Assay Kit, Resazurin based with indicator dye Alamar Blue (cat. TOX8, Sigma Alrich). Adherent and suspension cells were plated at 5×10^3 per well and $1x10^4$ per well respectively in triplicate in 96-well plates and incubated in medium containing 10% FBS. For adherent cells, the complete medium was replaced after 24 h and incubated with test medium containing vehicle control or serial concentration of compounds for 48 h at 37 °C. The remaining unused wells in the periphery of microtiter well plate were added with PBS to avoid evaporation effects. Alamar Blue was then added, and all plates were incubated at 37 °C, and a colorimetric change was measured according to the manufacturer's protocol.

Phospho-specific flow cytometry of intracellular protein

BaF3/ FLT3-ITD or K562 cells were seeded at 0.2 $x10^6$ cells ml^{-1} per well in 6-well plates overnight and incubated with serial concentration of tested compounds for 6 h. A total amount of 10^6 cells were collected and washed twice in 1xPBS and resuspended in 100 µl cytofix/cytoperm solution (cat. 554714, BD Biosciences) at 4 ºC. After 20 min, cells were washed twice with BD Perm/Wash buffer solution and incubated with 20 µl of specific fluorochrome (PE) conjugated monoclonal antibody anti-p-STAT5 (Phosflow™ PE-Cy™7 mouse anti-Stat5 (pY694) (BD Biosciences) at 4 ºC. After 30 min, the cells were washed twice and analyzed by flow cytometry. For isotype control, the cells were incubated with 2 µl of PE conjugated rat anti-mouse IgG1 monoclonal antibody (BD Biosciences) at 4 ºC for 30

min and washed twice in before being analyzed on a FACScan Flow Cytometer (Becton-Dickinson, San Jose, CA). Data interpretation was done using the Flowjo software (Treestar, Inc., San Carlos, CA)[22].

Cell viability assay

The effect of compounds on cell viability was evaluated using Cell Titer-Glo Luminescent Cell Viability assay kit (Promega). Cells were plated at 5×10^3 per well and $1x10^4$ per well respectively in triplicate in white-walled, clear-bottom 96-well plates (cat. 3903, Corning Costar) and incubated in medium containing 10% FBS overnight. Cells were treated with or without compounds at 37 ºC for 48 h before carrying out the viability assay. The number of viable cells was measured using the CellTiter-Glo ATP-dependent luminescent assay (Promega, Madison, WI) following the manufacturer instruction. Luminescence reading was measured using Tecan Infinite M1000 plate reader (Tecan, Männedorf, Switzerland). The graphically represented values are arithmetic means ± S.D. for three independent samples.

Cell apoptosis analysis

Cell apoptosis was determined using Annexin V staining. BaF3/FLT3-ITD or K562 suspension cells were plated at $0.2x10^6$ per well in 6-well plates and incubated with serial concentration of tested compounds for 48 h. Cells were washed twice in ice-cold PBS resuspended in 1x binding buffer (10 mM Hepes (pH 7.4), 140 mM NaCl and 2.5 mM $CaCl_2$) to a final concentration of $1x\ 10^6$ cells ml^{-1}. Subsequently, FITC Annexin V solution (5 µl) was added to 100 µl of the cell suspension. The mixture was incubated for 30 min at room temperature and washed in 1x Binding Buffer and again resuspended in 200 µl of 1x Binding buffer. Propidium iodide staining solution (5 µl, Sigma) was added shortly before analysis on a FACScan Flow Cytometer (Becton-Dickinson, San Jose, CA). Data interpretation was done using the Flowjo software (Treestar, Inc., San Carlos, CA).

General chemical methods.

Chemicals were purchased from Acros, Alfa Aesar, Fluka, Sigma-Aldrich, and VWR used without further purification or distillation unless otherwise stated. All solvents that were used during the reactions were obtained from a column-based solvent system (MBraun, MB-SPS-800). Solution phase reactions were monitored either by LC-MS techniques or by thin layer chromatography (TLC) using Analtech silica gel plates (60 F_{254}) and the spots were examined under UV light at 254 nm or stained with developing reagents. LC-MS data were recorded on an Agilent 1100 series chromatography workstation (Agilent Technologies) equipped with a single quadruple mass spectrometer and electrospray ionization (ESI). Eluents A (0.1 % formic Acid in Millipore water) and B (0.1 % formic Acid in acetonitrile) were used in a linear gradient (5-99 % B in 5 min or 30 min for preperative runs). Removal of solvents was performed using rotary evaporators from Heidolph. Product isolations were carried out on HPLC column (10 µM, 250 x 20 mm, Grom-SIL 300 ODS-5-ST RP-C18) or on a semi-preparative HPLC column (VP 250/10 Nucleodor 100-5 C18 ec Machery-Nagel) employing individual gradients derived from analytical runs (eluents A and B). Besides the Biotage® Isolera™ Spektra One was used for regular flash purifications with pre-pack flash chromatography cartrigdes from Biotage® employing individual gradients derived from analytical runs or thin layer chromatography (TLC). Product fractions were lyophilized from acetonitrile / water mixtures. Non-HRMS measurements were recorded with a ESI single quad spectrometer (Agilent) coupled with an analytical HPLC system (Agilent 1100). HRMS measurements were conducted with an Agilent 6210 ESI-TOF mass spectrometer. Nuclear magnetic resonance (NMR) spectra (^{1}H and ^{13}C NMR) were recorded on a Bruker AVANCE 300 MHz and 500 MHz instrument and chemical shifts (δ) were measured in parts per million (ppm). Coupling constants (*J*) are expressed in Hertz (Hz). The following abbreviations are used to describe peak patterns when appropriate: s (singlet), d (doublet), t (triplet), q (quadruplet), m (multiplet), and br (broad). The Biotage® Initator+

system was used for rapid microwave-assisted organic synthesis. The elemental analyzer VARIO EL was used for C-, H-, and N-analysis.

General synthetic methods

Method A: To a solution of 4-amino-furazan-3-carboxylic acid or methylester (1.0 mmol) and tetrazole (1.2 mmol) in 2.7 ml of acetonitrile (hipersolv chromanorm) and 0.3 ml of acetic acid, formaldehyde (37 % solution in water, 2.0 mmol) was added and this reaction mixture was stirred at r.t. for 16 h. Afterwards the mixture was lyophilized and the residue was purified by flash column chromatograph.

Method B: A solution of 4-amino-furazan-3-carboxylic acid (1.2 mmol), tetrazole (1.0 mmol), and formaldehyde (37 % aqueous solution, 10.0 mmol) in 2.7 ml of acetonitrile and 0.3 ml of concentrated hydrochloric acid was stirred in a sealed microwave reaction vial at 105 °C in a microwave reactor for 5 h. After cooling the reaction, solids were filtrated off, washed with a small amount of water and dried under reduced pressure. The residue was purified by flash column chromatography.

Method C: A suspension of the nitrile component (1.0 mmol), NaN_3 (2 mmol), and NH_4Cl (1.1 mmol) in DMF (5 ml) was stirred in a sealed microwave reaction vial at 140 °C in a microwave reactor for 1 h. After the mixture was evaporated in vacuo, the residue was poured into water and acidified with concentrated HCl to pH = 2 and cooled to 5 °C. Then the precipitate was filtrated off, washed with cold water, and dried under reduced pressure to give the desired compound. A reaction scheme for method C is shown as Supplementary Figure 12.

5-CF-GY*LSLPPW-NH_2 [1]

Synthesized as described previously.[17]

4-Formylphenyl-dihydrogen-phosphate [2]

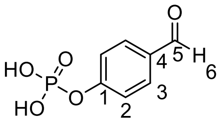

Diethyl chlorophosphate (1.18 ml, 8.19 mmol, 1 eq.) was added dropwise to a cooled (0°C) solution of 4-hydroxybenzaldehyde (1.0 g, 8.19 mmol, 1 eq.) and triethylamine (1.36 ml, 9.83 mmol, 1.2 eq.) in dry DCM (5 ml) under inert atmosphere. The reaction mixture was warmed to room temperature and stirred for 3 h. Afterwards the organic phase was extracted with 1 M HCl, saturated $NaHCO_3$, and dried over sodium sulfate. After filtration the filtrate was evaporated in vacuo. The di-O-ethyl-protected phosphate (0.5 g, 1.94 mmol, 1 eq.) was stirred with trimethylsilylbromide (0.51 ml, 3.88 mmol, 2 eq.) in MeCN (5 ml) at room temperature for 6 h. Subsequently, the reaction mixture was quenched with 10 ml of H_2O/MeOH (1:10), Amberlite resin IR120 (4 g) was added in protonated form and the mixture was stirred at room temperature for 12 h. After filtration and purification by flash column chromatography the product was obtained as a white solid (0.254 g, 1.26 mmol, 64 %).

^{1}H-NMR (500 MHz, DMSO-d_6): δ = 9.94 (s, 1H, H-6), 7.92 (d, J = 8.3 Hz, 2H, H-3), 7.37 (d, J = 8.3 Hz, 2H, H-2) ppm; **^{13}C-NMR** (101 MHz, DMSO-d_6): δ = 192.32 (C-5), 156.36 (C-1), 132.23 (C-4), 132.09(C-3), 120.94 (C-2) ppm; **^{31}P-NMR** (162 MHz, DMSO-d_6):δ = 21.3 (m, 1P, P) ppm; **HRMS:** (ESI): $C_7H_7O_5P$ [M], 202.0031 Da. calcd *m/z* 200.9953 $[M-H]^-$, found *m/z* 200.9959 $[M-H]^-$.

4-Amino-1,2,5-oxadiazole-3-carboxylic acid [3]

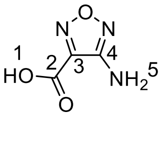

To a stirred suspension of ethyl 2-cyanoacetate (28.3 g, 0.25 mol, 1 eq.) and sodium nitrite (17.3 g, 0.25 mol, 1.0 eq.) in a mixture of EtOH (17 ml) and water (200 ml), 85% H_3PO_4 (10 ml) was added dropwise at 10-15 °C and stirred for 12 h. Afterwards, the reaction mixture was treated with NaOH (4×10 g, 1 mol, 4 eq.) and KOH (2×14 g, 0.5 mol, 2.0 eq.). To the resulting solution $NH_2OH\cdot HCl$ (69.5 g, 1.0 mol, 4.0 eq.) was added slowly at room temperature and heated to 95 °C, stirred for 2 h, cooled to ambient temperature and quenched with conc. HCl to pH 1. Precipitation occurred on cooling to 0 °C for 12 h and the precipitate was collected by filtration and dried. The filtrate was extracted with diethyl ether (3×30 ml). The combined organic extracts were evaporated under reduced pressure. The residue was combined with the precipitate and recrystallized from hot water to give compound **1** (21.3 g, 0.165 mol, 66 %) as a white solid.

^{1}H-NMR (300 MHz, DMSO-*d*$_6$): δ = 9.69 (br s, 1H, H-1), 6.24 (s, 2H, H-5) ppm; **^{13}C-NMR** (75 MHz, DMSO-*d*$_6$): δ = 162.5 (C-2), 157.1 (C-3), 144.9 (C-4) ppm; **HRMS:** (ESI): $C_3H_3N_3O_3$ [M], 129.0174 Da. calcd *m/z* 128.0096 $[M-H]^-$, found *m/z* 128.0098 $[M-H]^-$.

Methyl 4-amino-1,2,5-oxadiazole-3-carboxylate [X1]

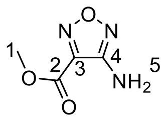

4-Amino-1,2,5-oxadiazole-3-carboxylic acid **3** (2 g, 15.5 mmol, 1.0 eq.) was dissolved in MeOH (20 ml) and a catalytic amount of conc. H_2SO_4 was added dropwise. The solution was heated to 60 °C and stirred for 3 h. Afterwards, the solvent was evaporated in vacuo. The residue was dissolved in DCM (50 ml), washed with water, a 1M solution of NaOH and brine. Subsequently, the solvent was evaporated and the remaining product was recrystallized from hot $CHCl_3$ to furnish compound **2** (2.11 g, 14.73 mmol, 95 %) as a white solid.

^{1}H-NMR (300 MHz, DMSO-*d*$_6$): δ = 6.24 (s, 2H, H-5), 3,76 (s, 3H, H-1) ppm; **^{13}C-NMR** (75 MHz, DMSO-*d*$_6$): δ = 159.4 (C-2), 156.5 (C-3), 139.9 (C-4), 53.5 (C-1) ppm;

HRMS:(ESI): $C_4H_5N_3O_3$ [M], 143.0331 Da. calcd *m/z* 166.0229 $[M+Na]^+$, 181.9968 $[M+K]^+$, found *m/z* 166.0233 $[M+Na]^+$, 181.9977 $[M+K]^+$.

4-Acetamido-1,2,5-oxadiazole-3-carboxylic acid [4]

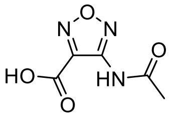

Compound **4** was available commercially at Sigma-Aldrich (#CDS002372).

Methyl 4-(2-bromoacetamido)-1,2,5-oxadiazole-3-carboxylate [X2]

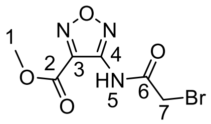

4-Amino-1,2,5-oxadiazole-3-carboxylic acid methylester **X1** (143 mg, 1 mmol, 1 eq.) and 4-DMAP (369.3 mg, 1.0 mmol, 1.0 eq.) were dissolved in dry DCM (15 ml) and 2-bromoacetyl bromide (242.2 mg, 1.2 mmol, 1.2 eq.) was slowly added under inert atmosphere. The reaction mixture was stirred at 0 °C for 2 h, evaporated in vacuo and purified by flash column chromatography to furnish product **X2** (339.7 mg, 0.92 mmol) in 92 % yield as a white solid. **^{1}H NMR** (300 MHz, $CDCl_3$): δ = 9.92 (s, 1H, H-5), 4.13 (s, 2H, H-7), 4.11 (s, 3H, H-1) ppm; **^{13}C-NMR** (75 MHz, $CDCl_3$): δ = 160.02 (C-6), 159.85 (C-2), 153.56 (C-3), 138.71 (C-4), 54.06 (C-1), 28.01 (C-7) ppm; **HRMS:** $C_6H_6BrN_3O_4$ [M], 262.9542 Da. calcd *m/z* 285.9439 $[M+Na]^+$, found *m/z* 285.9469 $[M+Na]^+$.

Methyl 4-(2-(*1H*-tetrazol-1-yl)-acetamido)-1,2,5-oxadiazole-3-carboxylate [X3]

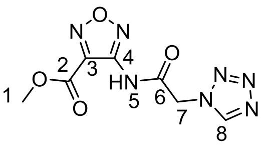

To a solution of methyl 4-(2-bromoacetamido)-1,2,5-oxadiazole-3-carboxylate **X2** (264.2 mg, 1.5 mmol, 1.0 eq.) and *1H*-tetrazol (9.1 ml (0.45M solution), 1.65 mmol, 1.1 eq.) in acetonitrile (5 ml), Et_3N (0.15 ml, 1.5 mmol, 1.0 eq.) was added and stirred at 90 °C for 5 h. After the mixture was evaporated in vacuo, the residue was dissolved in DCM (20 ml) and washed with H_2O (5 ml). The organic phase was dried over Na_2SO_4 and after filtration the filtrate was evaporated in vacuo. The residue was purified by flash column chromatography to furnish product **X3** (229.1 mg, 0.65 mmol) in 43 % yield as a white solid.

^{1}H NMR (300 MHz, CD_3CN): δ = 9.41 (s, 1H, H-5), 8.72 (s, 1H, H-8), 5.79 (s, 2H, H-7), 4.00 (s, 3H, H-1) ppm; **^{13}C-NMR** (75 MHz, CD_3CN): δ = 163.78 (C-6), 159.67 (C-2), 154.51 (C-3), 150.25 (C-8), 142.29 (C-4), 56.11 (C-1), 54.50 (C-7) ppm; **HRMS:** (ESI): $C_7H_7N_7O_4$ [M], 253.0560 Da. calcd *m/z* 276.0457 $[M+Na]^+$, found *m/z* 276.0451 $[M+Na]^+$.

4-(2-(1*H*-tetrazol-1-yl)-acetamido)-1,2,5-oxadiazole-3-carboxylic acid [5]

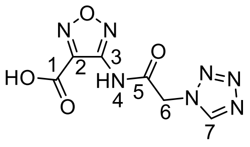

To a stirred solution of methyl 4-(2-(*1H*-tetrazol-1-yl)-acetamido)-1,2,5-oxadiazole-3-carboxylate **X3** (50.0 mg, 0.2 mmol, 1.0 eq.) in THF (1.0 ml), 0.1 M LiOH (0.1 ml) and water (0.4 ml) were added. The reaction mixture was stirred at r.t. for 30 min. Afterwards, the reaction mixture was neutralized with Amberlite IR-120 (H^+-form, filtrated and washed with H_2O/THF (2.0 ml, 1:1 (*v*/*v*)). The mixture was evaporated to give compound **5** (43.9 mg, 0.18 mmol, 92 %) as a white solid.

^{1}H NMR (300 MHz, CD_3CN): δ = 9.31 (s, 1H, H-4), 8.82 (s, 1H, H-7), 5.43 (s, 2H, H-6) ppm; **^{13}C-NMR** (75 MHz, CD_3CN): δ = 164.28 (C-5), 160.61 (C-1), 155.11 (C-2), 151.3 (C-7), 142.89 (C-3), 54.62 (C-6) ppm; **HRMS:** (ESI): $C_6H_5N_7O_4$ [M], 239.0403 Da. calcd *m/z* 262.0301$[M+Na]^+$, found *m/z* 262.0296 $[M+Na]^+$.

4-(*1H*-1,2,3-Triazol-1-yl)-methylamino-1,2,5-oxadiazole-3-carboxylic acid [6]

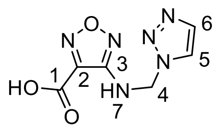

Prepared according to general procedure Method A using 4-amino-1,2,5-oxadiazole-3-carboxylic acid **3** (129 mg, 1 mmol, 1.0 eq.), *1H*-1,2,3-triazole (103.6, 1.5 mmol, 1.5 eq.) and formaldehyde (0.2 ml, 2.0 mmol, 2.0 eq.) to furnish product **6** (165.9 mg, 0.79 mmol) in 79 % yield as a white solid.

^{1}H NMR (300 MHz, DMSO-d_6): δ = 8.16 (m, 1H, H-6), 7.81 (t, *J* = 7.0 Hz, 1H, H-7), 7.71 (m, 1H, H-5), 5.82 (d, *J* = 6.9 Hz, 2H, H-7) ppm; **^{13}C-NMR** (75 MHz, DMSO-d_6): δ = 160.1 (C-1), 155.4 (C-2), 140.2 (C-3), 133.2 (C-6), 124.6 (C-5), 57.8 (C-4) ppm; **HRMS:** (ESI): $C_6H_6N_6O_3$ [M], 210.1530 Da. calcd *m/z* 209.1450 $[M-H]^-$, found *m/z* 209.1470 $[M-H]^-$.

4-(*1H*-Benzo[d]-1,2,3-triazol-1-yl)-methylamino-1,2,5-oxadiazole-3-carboxylic acid [7]

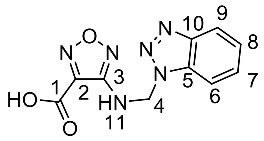

Prepared according to general procedure Method A using 4-amino-1,2,5-oxadiazole-3-carboxylic acid **3** (129 mg, 1.0 mmol, 1.0 eq.), *1H*-benzotriazole (179 mg, 1.5 mmol, 1.5 eq.) and formaldehyde (0.2 ml, 2.0 mmol, 2.0 eq.) to furnish product **7** (93.8 mg, 0.36 mmol) in 36 % yield as a white solid.

^{1}H NMR (300 MHz, DMSO-d_6): δ = 8.09 (d, *J* = 8.4 Hz, 1H, H-9), 8.05 (t,*J* = 7.0 Hz 1H, H-11), 8.03 (d, *J* = 8.3 Hz 1H, H-6), 7.57 (t, *J* = 7.6 Hz, 1H, H-8), 7.39 (t, *J* = 7.5 Hz, 1H, H-7), 6.17 (d, *J* = 6.9 Hz, 2H, H-4) ppm; **^{13}C-NMR** (75 MHz, DMSO-d_6): δ = 160.2 (C-1), 156.1 (C-2), 145.8 (C-10), 143.5 (C-3), 132.9 (C-5), 127.9 (C-8), 124.6 (C-7), 119.6(C-9), 111.9(C-

6), 57.2(C-4) ppm; **HRMS:** (ESI): $C_{10}H_8N_6O_3$ [M], 260.0658 Da. calcd *m/z* 259.0580 [M-H]$^-$, found *m/z* 259.0590 [M-H]$^-$.

4-(*1H*-Pyrazol-1-yl)-methylamino-1,2,5-oxadiazole-3-carboxylic acid [8]

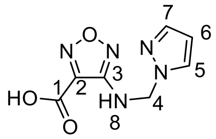

Prepared according to general procedure Method A using 4-amino-1,2,5-oxadiazole-3-carboxylic acid **3** (129 mg, 1.0 mmol, 1.0 eq.), *1H*-pyrazole (102.1, 1.5 mmol, 1,5 eq.) and formaldehyde (0.2 ml, 2.0 mmol, 2.0 eq.) to furnish product **8** (198.8 mg, 0.95 mmol) in 95 % yield as a white solid.

^{1}H NMR (300 MHz, DMSO-*d*$_6$): δ = 7.82 (d, *J* = 2.2 Hz, 1H, H-7), 7.61 (t, *J* = 6.9 Hz, 1H, H-8), 7.45 (d, *J* = 2.1 Hz, 1H, H-5), 6.23 (t, *J* = 2.1 Hz, 1H, H-6), 5.52 (d, *J* = 6.8 Hz, 3H, H-4) ppm; **^{13}C-NMR** (75 MHz, DMSO-*d*$_6$): δ = 159.7 (C-1), 155.6 (C-2), 139.1 (C-3), 130.1 (C-7), 129.5 (C-5), 105.3 (C-6), 59.2 (C-4) ppm; **HRMS:** (ESI): $C_7H_7N_5O_3$ [M], 209.0549 Da. calcd *m/z* 208.0471 [M-H]$^-$, found *m/z* 208.0465 [M-H]$^-$.

4-(*1H*-1,2,4-Triazol-1-yl)-methylamino-1,2,5-oxadiazole-3-carboxylic acid [9]

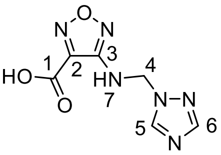

Prepared according to general procedure Method A using 4-amino-1,2,5-oxadiazole-3-carboxylic acid **3** (129 mg, 1.0 mmol, 1.0 eq.), *1H*-1,2,4-triazolezole (103.6, 1.5 mmol, 1.5 eq.) and formaldehyde (0.2 ml, 2.0 mmol, 2.0 eq.) to furnish product **9** (138.7 mg, 0.66 mmol) in 66 % yield as a white solid.

^{1}H NMR (300 MHz, DMSO-*d*$_6$): δ = 8.59 (s, 1H, H-6), 7.97 (s, 1H, H-5), 7.69 (t, *J* = 6.9 Hz, 1H, H-7), 5.61 (d, *J* = 6.8 Hz, 2H, H-4) ppm; **^{13}C-NMR** (75 MHz, DMSO-*d*$_6$): δ = 159.6 (C-

1), 155.3 (C-2), 151.5 (C-3), 144.5 (C-5), 140.2 (C-6), 57.3 (C-4) ppm; **HRMS:** (ESI): $C_6H_6N_6O_3$ [M], 210.1530 Da. calcd *m/z* 209.1450 $[M-H]^-$, found *m/z* 209.1459 $[M-H]^-$.

4-(*1H*-Tetrazol-1-yl)-methylamino-1,2,5-oxadiazole-3-carboxylic acid [10]

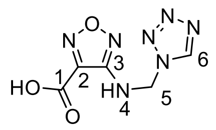

Prepared according to general procedure Method A using 4-amino-1,2,5-oxadiazole-3-carboxylic acid **3** (129 mg, 1.0 mmol, 1.0 eq.), *1H*-tetrazol (3.3 ml (0.45M solution), 1.5 mmol, 1.5 eq.) and formaldehyde (0.2 ml, 2.0 mmol, 2.0 eq.) to furnish product **10** (184.8 mg, 0.88 mmol) in 88 % yield as a white solid.

^{1}H NMR (300 MHz, DMSO-d_6):δ = 9.43 (s, 1H, H-6), 7.90 (t, *J* = 6.9 Hz, 1H, H-4), 5.91 (d, *J* = 6.9 Hz, 2H, H-5) ppm; **^{13}C-NMR** (75 MHz, DMSO-d_6): δ = 160.2 (C-1), 155.8 (C-2), 144.6 (C-3), 140.7 (C-6), 57.2 (C-5) ppm; **HRMS:**(ESI): $C_5H_5N_7O_3$ [M], 211.0454 Da. calcd *m/z* 210.0376 $[M-H]^-$, found *m/z* 210.0369 $[M-H]^-$. **Anal:** calcd for $C_5H_5N_7O_3$: C, 28.44; H, 2.39; N, 46.44; found C, 28.12; H, 2.26; N, 46.82.

4-(5-Phenyl-*1H*-tetrazol-1-yl)-methylamino-1,2,5-oxadiazole-3-carboxylic acid [11]

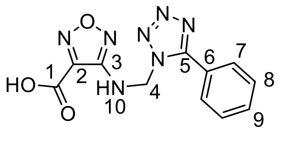

Prepared according to general procedure Method A using 4-amino-1,2,5-oxadiazole-3-carboxylic acid **3** (129 mg, 1.0 mmol, 1.0 eq.), 5-phenyl-*1H*-tetrazole (219 mg, 1.5 mmol, 1.5 eq.) and formaldehyde (0.2 ml, 2.0 mmol, 2.0 eq.) to furnish product **11** (252.6 mg, 0.88 mmol) in 88 % yield as a white solid.

^{1}H NMR (300 MHz, DMSO-d_6): δ = 8.10 (t, *J* = 7.0 Hz, 1H, H-10), 8.04 (d, *J* = 7.8 Hz, 2H, H-7), 7.58 – 7.52 (m, 3H, H-8, H-9), 6.14 (d, *J* = 7.0 Hz, 2H, H-4) ppm; **^{13}C-NMR** (75 MHz,

DMSO-d_6): δ = 164.7 (C-5), 160.2 (C-1), 155.8 (C-2), 140.7 (C-3), 131.2 (C-6), 129.9 (C-7), 127.4 (C-9), 126.9 (C-8), 62.1 (C-4) ppm; **HRMS:** (ESI): $C_{11}H_9N_7O_3$ [M], 287.2390 Da. calcd *m/z* 286.2310 $[M-H]^-$, found *m/z* 286.2319 $[M-H]^-$.

4-(5-(3-(Trifluoromethyl)-phenyl)-*1H*-tetrazol-1-yl)-methylamino-1,2,5-oxadiazole-3-carboxylic acid [12]

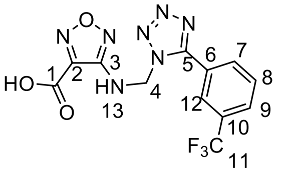

Prepared according to general procedure Method A using 4-amino-1,2,5-oxadiazole-3-carboxylic acid **3** (83.9 mg, 0.7 mmol, 1.3 eq.), 5-(4-(trifluoromethyl)phenyl)-*1H*-tetrazole (147.2 mg, 0.5 mmol, 1.0 eq.) and formaldehyde (0.1 ml, 2.0 mmol, 2.0 eq.) to furnish product **12** (159.9 mg, 0.45 mmol) in 90 % yield as a white solid. **^{1}H NMR** (300 MHz, $CDCl_3$): δ = 8.40 (s, 1H, H-12), 8.32 (d, *J* = 7.8 Hz, 1H, H-7), 7.72 (d, *J* = 7.9 Hz, 1H, H-9), 7.60 (t, *J* = 7.8 Hz, 1H, H-8), 6.88 (t, *J* = 7.5 Hz, 1H, H-13), 6.20 (d, *J* = 7.6 Hz, 2H, H-4) ppm; **^{13}C-NMR** (75 MHz, $CDCl_3$): δ = 163.6 (C-5), 160.90 (C-1), 155.20 (C-2), 138.94 (C-3), 131.68(C-10), 130.16 (C-7), 129.47 (C-8), 127.83 (C-6), 127.06 (C-9), 125.54 (C-11), 123.87 (C-12), 60.72 (C-4) ppm; **HRMS:** (ESI): $C_{12}H_8F_3N_7O_3$ [M], 355.0641 Da. calcd *m/z* 394.0278 $[M+K]^+$, found *m/z* 394.0263$[M+K]^+$.

4-(5-(3-Fluorophenyl)-*1H*-tetrazol-1-yl)-methylamino-1,2,5-oxadiazole-3-carboxylic acid [13]

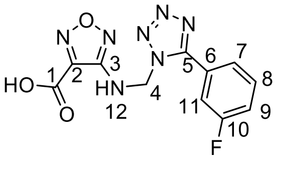

Prepared according to general procedure Method B using 4-amino-1,2,5-oxadiazole-3-carboxylic acid **3** (167.7 mg, 0.7 mmol, 1.3 eq.), 5-(3-fluorophenyl)-*1H*-tetrazole (164.2 mg, 1.0 mmol, 1.0 eq.) and formaldehyde (0.15 ml, 2.0 mmol, 2.0 eq.) to furnish product **13** (250.3 mg, 0.82 mmol) in 82 % yield as a white solid.

^{1}H NMR (300 MHz, $CDCl_3$): δ = 7.94 (d, *J* = 7.8 Hz, 1H, H-7), 7.84 (dd, *J* = 9.5, 2.2 Hz, 1H, H-11), 7.46 (ddd, *J* = 8.3, 7.8, 5.8 Hz, 1H, H-8), 7.17 (t, *J* = 8.4 Hz, 1H, H-9), 6.85 (t, *J* = 7.6 Hz, 1H, H-12), 6.20 (d, *J* = 7.6 Hz, 2H, H-4) ppm; **^{13}C-NMR**(75 MHz, DMSO-d_6): δ = 163.72 (C-5), 159.56 (C-1), 155.83 (C-2), 140.95 (C-3), 134.28 (C-6), 131.65 (C-10), 129.16 (C-8), 123.72 (C-7), 121.85 (C-11), 118.93 (C-9), 61.85 (C-4) ppm; **HRMS:** (ESI): $C_{11}H_8FN_7O_3$ [M], 305.0673 Da. calcd *m/z* 304.0594 $[M-H]^-$, found *m/z* 304.0593 $[M-H]^-$.

4-(5-(3-(Trifluoromethoxy)-phenyl)-1*H*-tetrazol-1-yl)-methylamino-1,2,5-oxadiazole-3-carboxylic acid [14]

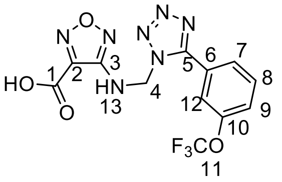

Prepared according to general procedure Method B using 4-amino-1,2,5-oxadiazole-3-carboxylic acid **3** (83.9 mg, 0.7 mmol, 1.3 eq.), 3-(1H-tetrazol-5-yl)phenyl hypofluorite (115.1 mg, 1.0 mmol, 1.0 eq.) and formaldehyde (0.1 ml, 2.0 mmol, 2.0 eq.) to furnish product **14** (252.4 mg, 0.82 mmol) in 68 % yield as a white solid.

^{1}H NMR (300 MHz, $CDCl_3$): δ = 8.09 (d, *J* = 8.0 Hz, 1H, H-7), 8.00 (s, 1H, H-12), 7.52 (dd, *J* = 7.8 Hz, 1H, H-8), 7.32 (d, *J* = 8.0 Hz, 1H, H-9), 6.85 (t, *J* = 7.6 Hz, 1H, H-13), 6.20 (d, *J* = 7.6 Hz, 2H, H-4) ppm; **^{13}C-NMR** (75 MHz, DMSO-d_6): δ = 163.20 (C-5), 159.82 (C-1), 155.54 (C-2), 149.11 (C-10), 140.82 (C-3), 132.03 (C-8), 129.21 (C-6), 126.32 (C-7), 125.76 (C-9), 123.44 (C-11), 118.73 (C-12), 62.04 (C-4) ppm; **HRMS:** (ESI): $C_{12}H_8F_3N_7O_4$ [M], 371.2362 Da. calcd *m/z* 370.2282 $[M-H]^-$, found *m/z* 370.2247 $[M-H]^-$.

4-(5-(4-Fluorophenyl)-*1H*-tetrazol-1-yl)-methylamino-1,2,5-oxadiazole-3-carboxylic acid [15]

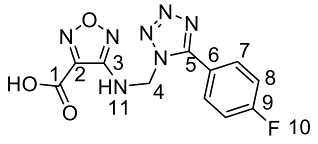

Prepared according to general procedure Method B using 4-amino-1,2,5-oxadiazole-3-carboxylic acid **3** (167.7 mg, 0.7 mmol, 1.3 eq.), 5-(4-fluorophenyl)-1H-tetrazole (164.2 mg, 1.0 mmol, 1.0 eq.) and formaldehyde (0.15 ml, 2.0 mmol, 2.0 eq.) to furnish product **15** (213.7 mg, 0.7 mmol) in 70 % yield as a white solid. **^{1}H NMR** (300 MHz, $CDCl_3$): δ = 8.14 (dd, *J* = 8.7, 5.5 Hz, 2H, H-7), 7.17 (t, *J* = 8.7 Hz, 2H, H-8), 6.81 (t, *J* = 7.6 Hz, 1H, H-11), 6.19 (d, *J* = 7.6 Hz, 2H, H-4) ppm; **^{13}C-NMR** (75 MHz, DMSO-*d*$_6$): δ = 163.68 (C-5), 159.83 (C-1), 155.56 (C-2), 140.61 (C-3), 134.08 (C-6), 129.85 (C-7), 129.16 (C-8), 123.72 (C-9), 116.85 (C-10), 61.85 (C-4) ppm; **HRMS:** (ESI): $C_{11}H_8FN_7O_3$ [M], 305.0673 Da. calcd *m/z* 304.0594 $[M\text{-}H]^-$, found *m/z* 304.0599 $[M\text{-}H]^-$.

4-(5-Benzyl-*1H*-tetrazol-1-yl)-methylamino-1,2,5-oxadiazole-3-carboxylic acid [16]

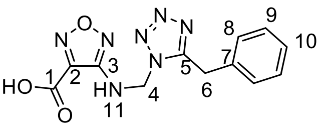

Prepared according to general procedure Method B using 4-amino-1,2,5-oxadiazole-3-carboxylic acid **3** (80.2 mg, 0.62 mmol, 1.2 eq.), 5-benzyl-1H-tetrazole (100.0 mg, 0.52 mmol, 1.0 eq.) and formaldehyde (0.47 ml, 5.2 mmol, 10.0 eq.) to furnish product **16** (81.3 mg, 0.27 mmol) in 52 % yield as a off white solid.

^{1}H NMR (500 MHz, DMSO-*d*$_6$): δ = 7.9 (t, *J* = 6.6 Hz, 2H, H-11), 7.27 (d, *J* = 7.0 Hz, H-8), 7.25 - 7.22 (m, 3H, H-9, H-10), 5.89 (d, *J* = 6.6 Hz, 2H, H-4), 4.48 (s, 2H, H-6) ppm; **^{13}C-NMR** (126 MHz, DMSO-*d*$_6$): δ = 159.99 (C-1), 155.42 (C-2), 154.9 (C-5), 140.28 (C-3),

135.65 (C-7), 129.17 (C-8), 128.93 (C-9), 127.34 (C-10), 56.36 (C-4), 28.57 (C-6) ppm; **HRMS:** (ESI): $C_{12}H_{11}N_7O_3$ [M], 301.0923 Da. calcd *m/z* 300.0845 $[M-H]^-$, found *m/z* 300.0805 $[M-H]^-$. **Anal:** calcd for $C_{12}H_{11}N_7O_3$: C, 47.84; H, 3.68; N, 32.55; found C, 48.11; H, 3.81; N, 32.18.

4-(5-([1,1'-Biphenyl]-4-yl)-1*H*-tetrazol-1-yl)-methylamino-1,2,5-oxadiazole-3-carboxylic acid [17]

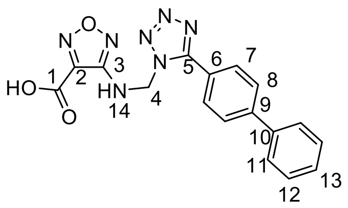

Prepared according to general procedure Method A using 4-amino-1,2,5-oxadiazole-3-carboxylic acid **3** (143 mg, 1.0 mmol, 1.0 eq.), 5-([1,1'-biphenyl]-4-yl)-1*H*-tetrazole (266 mg, 1.2 mmol, 1.2 eq.) and formaldehyde (0.1 ml, 2.0 mmol, 2 eq.) to furnish product **17** (221.5mg, 0.61 mmol) in 61 % yield as a white solid.

^{1}H NMR (300 MHz, $CDCl_3$): δ = 8.14 (d, *J* = 8.2 Hz, 2H, H-7), 8.11 (t, *J* = 7.0 Hz, 1H, H-14), 7.86 (d, *J* = 8.4 Hz, 2H, H-8), 7.74 (d, *J* = 7.2 Hz, 2H, H-11), 7.50 (t, *J* = 7.6 Hz, 2H, H-12), 7.41 (t, *J* = 7.2 Hz, 1H, H-13), 6.17 (d, *J* = 7.0 Hz, 2H, H-4) ppm; **^{13}C-NMR** (75 MHz, $CDCl_3$): δ = 164.41 (C-5), 160.09 (C-1), 155.78 (C-2), 142.59 (C-9), 140.69 (C-10), 139.58 (C-3), 129.54 (C-12), 128.52 (C-13), 127.95 (C-6), 127.45 (C-7), 127.20 (C-8), 126.27 (C-11), 62.03 (C-4) ppm; **HRMS:** (ESI): $C_{17}H_{13}N_7O_3$ [M], 363.1080 Da. calcd *m/z* 386.0978 $[M+Na]^+$, found *m/z* 386.0970 $[M+Na]^+$.

Methyl 4-(*1H*-tetrazol-1-yl)-methylamino-1,2,5-oxadiazole-3-carboxylate [18]

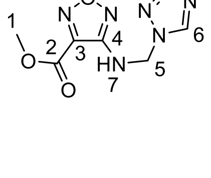

Prepared according to general procedure Method A using 4-amino-1,2,5-oxadiazole-3-carboxylic acid methylester **X1** (143 mg, 1.0 mmol, 1.0 eq.), *1H*-tetrazol (3.3 ml (0.45M solution), 1.5 mmol, 1.5 eq.) and formaldehyde (0.2 ml, 2.0 mmol, 2.0 eq.) to furnish product **18** (184.8 mg, 0.88 mmol) in 88 % yield as a white solid.

^{1}H NMR (300 MHz, DMSO-d_6): δ = 9.44 (s, 1H, H-6), 8.01 (t, J = 6.9 Hz, 1H, H-7), 5.92 (d, J = 6.9 Hz, 2H, H-5) ppm; **^{13}C-NMR** (75 MHz, DMSO-d_6): δ = 159.5 (C-2), 155.6 (C-3), 144.6 (C-4), 126.2 (C-6), 53.9 (C-5), 53.8 (C-1) ppm; **HRMS:** (ESI): $C_6H_7N_7O_3$ [M], 225.0610 Da. calcd *m/z* 248.0508 $[M+Na]^+$, found *m/z* 248.0510 $[M+Na]^+$.

1-Bromoethyl-acetate [X4]

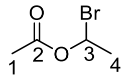

To a stirred solution of acetylbromide (0.85 ml, 11.35 mmol, 3.0 eq.) and catalytic amount of zinc(II)chloride (5 mg) in dry DCM (2.5 ml) was added paraformaldehyde (0.51 ml, 3.78 mmol, 1 eq.) under inert atmosphere and the reaction stirred for 45 min at 0 °C. The reaction mixture was quenched with water and the organic phase was washed two times with water. After evaporation of methylene chloride the product was obtained as colourless oil (467.8 mg, 2.82 mmol) in 75 %.

^{1}H NMR (400 MHz, DMSO-d_6): δ = 6.67 (q, J = 5.9 Hz, 1H, H-3), 2.09 (s, 3H, H-1)), 1.97 (d, J = 5.9 Hz, 3H, H-4) ppm; **^{13}C-NMR** (101 MHz, DMSO-d_6): δ = 168.45 (C-2), 71.76 (C-3), 26.82 (C-1), 21.06 (C-4) ppm; **HRMS:** (ESI): $C_4H_7BrO_2$ [M], 165.9629 Da. calcd *m/z* 166.9708 $[M+H]^+$, found *m/z* 166.9711 $[M+H]^+$.

1-Acetoxyethyl 4-(1*H*-tetrazol-1-yl-methylamino)-1,2,5-oxadiazole-3-carboxylate [19]

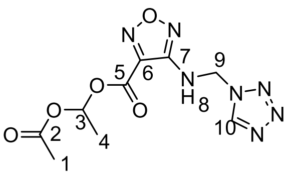

To a stirred solution of 4-(*1H*-tetrazol-1-yl-methylamino)-1,2,5-oxadiazole-3-carboxylic acid **16** (100 mg, 0.47 mmol, 1.0 eq.) and DIPEA (0.16 ml, 0.94 mmol, 2 eq.) in DMF (2 ml) was added 1-bromoethyl-acetate **X4** (156 mg, 0.94 mmol, 2 eq.) and the reaction was stirred for 18 h at room temperature. The reaction mixture was evaporated in vacuo and purified by flash column chromatography to furnish product **19** (63.7 mg, 0.21 mmol) in 45 % yield as a brownish oil.

^{1}H NMR (500 MHz, DMSO-*d*$_6$): δ = 9.44 (s, 1H, H-10), 8.02 (t, *J* = 6.9 Hz, 1H, H-8), 7.04 (q, *J* = 5.5 Hz, 1H, H-3), 5.93 (d, *J* = 6.9 Hz, 2H, H-9), 2.09 (s, 3H, H-1), 1.57 (d, *J* = 5.5 Hz, 3H, H-4) ppm; **^{13}C-NMR** (126 MHz, DMSO-*d*$_6$): δ = 169.03 (C-5), 156.90 (C-2), 155.76 (C-7), 144.48 (C-6), 139.22 (C-10), 89.85 (C-3), 56.97 (C-9), 20.98 (C-4), 20.75 (C-1) ppm; **HRMS:** (ESI): $C_9H_{11}N_7O_5$ [M], 297.0822 Da. calcd *m/z* 320.0719 $[M+Na]^+$, 336.0459 $[M+K]^+$, found *m/z* 320.0730 $[M+Na]^+$, 336.0470 $[M+K]^+$.

4-(5-(3-Benzyloxy-phenyl)-1H-tetrazol-1-yl)-methylamino-1,2,5-oxadiazole-3-carboxylic acid [20]

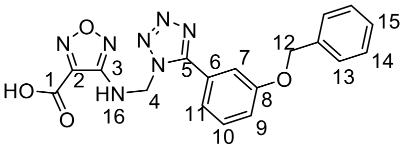

Prepared according to general procedure Method A using 4-amino-1,2,5-oxadiazole-3-carboxylic acid **3** (143 mg, 1.0 mmol, 1.0 eq.), 5-(3-benzyloxyphenyl)-*1H*-tetrazole (302 mg, 1.2 mmol, 1.2 eq.) and formaldehyde (0.1 ml, 2.0 mmol, 2 eq.) to furnish product **20** (204.24mg, 0.52 mmol) in 52 % yield as a white solid.

^{1}H NMR (300 MHz, $CDCl_3$): δ = 8.12 (t, *J* = 7.0 Hz, 1H, H-16), 7.67 – 7.62 (m, 2H, H-9, H-10), 7.49 (m, 1H, H-11), 7.48 (m, 2H, H-14), 7.40 (m, 2H, H-13), 7.34 (s, 1H, H-7), 7.19 (d, *J* = 8.4 Hz, 1H, H-15), 6.15 (d, *J* = 7.1 Hz, 2H, H-4), 5.20 (s, 2H, H-12) ppm; **^{13}C-NMR** (75 MHz, $CDCl_3$): δ = 164.47 (C-5), 160.06 (C-1), 159.27 (C-2), 155.78 (C-8), 137.27 (C-3), 131.06 (C-10), 128.93 (C-14), 128.58 (C-15), 128.35 (C-6), 128.23 (C-11), 128.15 (C-13), 119.41 (C-9), 117.83 (C-7), 69.82 (C-12), 58.60 (C-4) ppm; **HRMS:** (ESI): $C_{18}H_{15}N_7O_4$ [M], 393.1186 Da. calcd *m/z* 392.1107 $[M-H]^-$, found 392.1122 $[M-H]^-$.

Methyl 4-(5-phenyl-*1H*-tetrazol-1-yl)-methylamino-1,2,5-oxadiazole-3-carboxylate [21]

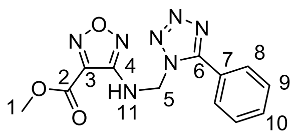

Prepared according to general procedure Method A using 4-amino-1,2,5-oxadiazole-3-carboxylic acid methylester **X1** (143 mg, 1.0 mmol, 1.0 eq.), 5-phenyl-*1H*-tetrazole (219 mg, 1.5 mmol, 1.5 eq.) and formaldehyde (0.2 ml, 2.0 mmol, 2 eq.) to furnish product **21** (183.7 mg, 0.61 mmol) in 61 % yield as a white solid.

^{1}H NMR (300 MHz, $CDCl_3$): δ = 8.13 (dd, *J* = 6.7, 2.9 Hz, 2H, H-9), 7.49 (m, 1H, H-10), 7.47 (d, *J* = 2.7 Hz, 2H, H-8), 6.69 (t, *J* = 7.5 Hz, 1H, H-11), 6.19 (d, *J* = 7.5 Hz, 2H, H-5), 4.03 (s, 3H, H-1) ppm; **^{13}C-NMR** (75 MHz, $CDCl_3$): δ = 165.7 (C-6), 159.9 (C-2), 155.2 (C-3), 137.9 (C-4) 130.6 (C-10), 128.9 (C-9), 126.9 (C-8), 126.9 (C-7), 60.4 (C-1), 53.6 (C-5) ppm; **HRMS:** (ESI): $C_{12}H_{11}N_7O_3$ [M], 301.2660 Da. calcd *m/z* 324.2558 $[M+Na]^+$, 340.3643 $[M+K]^+$, found *m/z* 324.2546 $[M+Na]^+$, 340.3648 $[M+K]^+$.

Methyl 4-(5-(3-hydroxyphenyl)-*1H*-tetrazol-1-yl)-methylamino-1,2,5-oxadiazole-3-carboxylate [22]

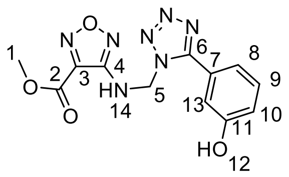

Prepared according to general procedure Method A using 4-amino-1,2,5-oxadiazole-3-carboxylic acid methylester **X1** (72.6 mg, 0.5 mmol, 1.0 eq.), 5-(3-hydroxyphenyl)-3-(*1H*-tetrazol, obtained using method C, (97.3 mg, 0.6 mmol, 1.2 eq.) and formaldehyde (0.1 ml, 1.0 mmol, 2.0 eq.) to furnish product **22** (236.2 mg, 0.64 mmol) in 64 % yield as a white solid.

^{1}H NMR (300 MHz, DMSO-d_6): δ = 9.82 (s, 1H, H-12), 8.18 (t, *J* = 6.9 Hz, 1H, H-14), 7.48 (m, 1H, H-10), 7.46 (s, 1H, H-13), 7.34 (t, *J* = 7.8 Hz, 1H, H-9), 6.91 (d, *J* = 8.1 Hz, 1H, H-8), 6.13 (d, *J* = 6.9 Hz, 2H, H-5), 3.94 (s, 3H, H-1); **^{13}C-NMR** (75 MHz, DMSO-d_6): δ = 164.79 (C-6), 158.81 (C-2), 158.6 (C-11), 155.61 (C-3), 140.03 (C-4), 131.05 (C-7), 128.44 (C-9), 118.25 (C-8), 117.61 (C-13), 113.47 (C-10), 61.93 (C-1), 53.81 (C-5); **HRMS:** (ESI): $C_{12}H_{11}N_7O_4$ [M], 317.0873 Da. calcd *m/z* 340.0770 $[M+Na]^+$, found *m/z* 340.0782 $[M+Na]^+$

Methyl 4-(5-(3-trifluoromethoxy-phenyl)-*1H*-tetrazol-1-yl)-methylamino-1,2,5-oxadiazole-3-carboxylate [23]

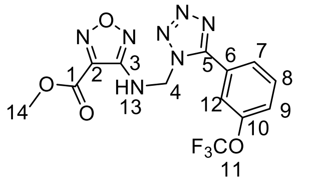

Prepared according to general procedure Method B using 4-amino-1,2,5-oxadiazole-3-carboxylic acid methylester (83.9 mg, 0.7 mmol, 1.3 eq.), 5-(3-trifluoromethoxy-phenyl)-1*H*-tetrazol, obtained by using method C, (115.1 mg, 1.0 mmol, 1.0 eq.) and formaldehyde (0.1 ml, 2.0 mmol, 2.0 eq.) to furnish product **23** (252.4 mg, 0.82 mmol) in 68 % yield as a yellowish solid.

^{1}H NMR (300 MHz, $CDCl_3$): δ = 8.22 (t, *J* = 7.8 Hz, 1H, H-8), 8.08 (d, *J* = 7.8 Hz, 1H, H-7), 7.91 (s, 1H, H-12), 7.72 (t, *J* = 7.5 Hz, 1H, H-13), 7.56 (d, *J* = 7.8 Hz, 1H), 6.17 (d, *J* = 7.3 Hz, 2H, H-4), 3.94 (s, 3H, H-14) ppm; **^{13}C-NMR** (75 MHz, DMSO-d_6): δ = ^{13}C NMR (126 MHz, DMSO-D_6) δ 163.49 (C-5), 158.80 (C-1), 155.58 (C-2), 149.41 (C-10), 140.03 (C-3), 132.34 (C-8), 129.45 (C-6), 126.03 (C-7), 123.75 (C-9), 122.39 (C-11), 118.98 (C-12), 62.23 (C-4), 53.80 (C-14) ppm; **HRMS:** (ESI): $C_{13}H_{10}F_3N_7O_4$ [M], 385.2632 Da. calcd *m/z* 408.2530 $[M+Na]^+$, 424.3615 $[M+K]^+$, found *m/z* 408.2524 $[M+Na]^+$, 424.3622 $[M+K]^+$.

Methyl 4-(5-(3-methylsulfonyl-amino-phenyl)-1*H*-tetrazol-1-yl-methylamino)-1,2,5-oxadiazole-3-carboxylate [24]

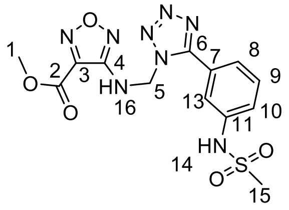

Prepared according to general procedure Method A using 4-amino-1,2,5-oxadiazole-3-carboxylic acid methylester **X1** (72.6 mg, 0.5 mmol, 1.0 eq.), 5-(3-methylsulfonyl-amino-phenyl)-1*H*-tetrazole (97.3 mg, 0.6 mmol, 1.2 eq.) and formaldehyde (0.1 ml, 1.0 mmol, 2.0 eq.) to furnish product **24** (236.2 mg, 0.64 mmol) in 64 % yield as a white solid.

^{1}H NMR (300 MHz, CD_3CN): δ = 7.98 (s, 1H, H-13), 7.89 (d, *J* = 7.6 Hz, 1, H-8), 7.70 (s, 1H, H-14), 7.51 (t, *J* = 7.9 Hz, 1H, H-9), 7.38 (d, *J* = 7.7 Hz, 1H, H-10), 7.09 (t, *J* = 7.3 Hz, 1H, H-16), 6.14 (d, *J* = 7.3 Hz, 2H, H-5), 3.98 (s, 3H, H-1), 2.97 (s, 3H, H-15) ppm; **^{13}C-NMR** (75 MHz, CD_3CN): δ = 165.48 (C-6), 160.10 (C-2), 156.44 (C-3), 140.38 (C-11), 139.80 (C-4), 131.41 (C-9), 129.58 (C-7), 123.76 (C-10), 123.18 (C-8), 119.10 (C-13), 61.98 (C-7), 54.13 (C-8), 39.78 (C-15) ppm; **HRMS:** (ESI): $C_{13}H_{14}N_8O_5S$ [M], 394.0808 Da. calcd *m/z* 417.0706 $[M+Na]^+$, found *m/z* 417.0706 $[M+Na]^+$.

1*H*-Tetrazole [25]

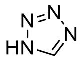

Commercially available at Sigma-Aldrich (#88185), lyophilization of the 0.45 M solution yielded 1*H*-tetrazol as a white solid.

5-Benzyl-1*H*-tetrazole [26]

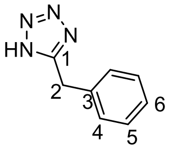

Prepared according to general procedure Method C using phenylacetonitrile (1.15 ml, 10.0 mmol, 1.0 eq.), NaN_3 (1.3 g, 20.0 mmol, 2.0 eq.), and NH_4Cl (535 mg, 10.0 mmol, 1.0 eq.) to furnish product **26** (1039.7 mg, 0.64 mmol) in 65 % yield as an off white solid. **^{1}H NMR** (500 MHz, DMSO-*d*$_6$): δ = 7.39 - 7.29 (m, 2H, H-5), 7.33 - 7.27 (m, 2H, H-4), 7.26 - 7.22 (m, 1H, H6), 4.29 (s, 2H, H-2) ppm; **^{13}C-NMR** (126 MHz, DMSO-*d*$_6$): δ = 145.09 (C-1), 138.20 (C-3), 129.28 (C-5), 129.21 (C-4), 127.57 (C-6), 33.87 (C-2) ppm; **HRMS:** (ESI): $C_8H_8N_4$ [M], 160.0749 Da. calcd *m/z* 183.0647 $[M+Na]^+$, found *m/z* 183.0651 $[M+Na]^+$.

5-CF -K(biotin)GpcFLSLPPW-NH$_2$ [27]

The dual labeled peptide **27** was synthesized using the photoactivatable building block sodium *N*-fluorenyl-9-methyloxycarbonyl-4-(*O*-benzyl-sodiumphosphonocarbonyl)-phenylalanine (Fmoc-pcF-OH), which was provided by a multi-step protocol.[17] For the evaluation of photo-crosslinking experiments utilizing avidin-biotin analysis *N*-fluorenyl-9-methoxycarbonyl-*N*^{6}-(biotinyl)-lysine was synthesized and incorporated.[23] Coupling of *N*-Fmoc-protected amino acids and consequent basic deprotection that followed the photoactive building block were performed using protocols described in literature[24]. Acylation with 5,6-carboxyfluorescein, purification with HPLC buffered with NH_4HCO_3 and ion-exchange with

sodium salt furnished peptide **27** in 12 % yield. **HRMS** (ESI-TOF, [m/z]): calculated: $[M+H^+]^+$: 1733.6757; found $[M+H^+]^+$: 1733.6907.

Supplementary References

1. Nikolovska-Coleska, Z. *et al.* Development and optimization of a binding assay for the XIAP BIR3 domain using fluorescence polarization. *Anal. Biochem.* **332,** 261-273 (2004).
2. Hopkins, A. L., Keseru, G. M., Leeson, P. D., Rees, D. C. & Reynolds, C. H. The role of ligand efficiency metrics in drug discovery. *Nat. Rev. Drug Discov.* **13,** 105-121 (2014).
3. Schust, J., Sperl, B., Hollis, A., Mayer, T. U. & Berg, T. Stattic: A small-molecule inhibitor of STAT3 activation and dimerization. *Chem. Biol.* **13,** 1235-1242 (2006).
4. Krishna, S. N. *et al.* A fluorescence-based thermal shift assay identifies inhibitors of mitogen activated protein kinase kinase 4. *PLoS ONE* **8 (12),** e81504 (2013).
5. Elumalai, N., Berg, A., Natarajan, K., Scharow, A. & Berg, T. Nanomolar inhibitors of the transcription factor STAT5b with high selectivity over STAT5a. *Angew. Chem. Int. Ed.* **54,** 4758-4763 (2015).
6. Hopkins, A. L., Keseru, G. M., Leeson, P. D., Rees, D. C. & Reynolds, C. H. The role of ligand efficiency metrics in drug discovery. *Nat. Rev. Drug Discov.* **13,** 105-121 (2014).
7. Neculai, D. *et al.* Structure of the Unphosphorylated STAT5a Dimer. *J. Biol. Chem.* **280,** 40782-40787 (2005).
8. Lin, J.X., Mietz, J., Modi, W. S., John, S. & Leonard, W. J. Cloning of human Stat5B reconstitution of interleukin-2-induced Stat5A and Stat5B DNA binding activity in COS-7 cells. *J. Biol. Chem.* **271,** 10738-10744 (1996).
9. SYBYL, X. Tripos International, 1699 South Hanley Rd. *St. Louis, Missouri, 63144, USA*.

10 Lunney, E. A. *et al.* Structure-based design of a novel series of nonpeptide ligands that bind to the pp60src SH2 domain. *J. Am. Chem. Soc.* **119,** 12471-12476 (1997).

11 Lange, G. *et al.* Requirements for specific binding of low affinity inhibitor fragments to the SH2 domain of pp60Src are identical to those for high affinity binding of full length inhibitors. *J. Med. Chem.* **46,** 5184-5195 (2003).

12 Morris, Garrett M. *et al.* AutoDock4 and AutoDockTools4: Automated docking with selective receptor flexibility. *J. Comput. Chem.* **30 (16),** 2785–2791 (2009).

13 Trott, O., & Olson, A. J. AutoDock Vina: improving the speed and accuracy of docking with a new scoring function, efficient optimization, and multithreading. *J. Comput. Chem.* **31 (2),** 455-461 (2010).

14 Durrant, J. D., & McCammon, J. A. BINANA: A novel algorithm for ligand-binding characterization. *J. Mol. Graph. Model.* **29 (6),** 888–893 (2011).

15 LaRochelle, Jonathan R. *et al.* Identification of an allosteric benzothiazolopyrimidone inhibitor of the oncogenic protein tyrosine phosphatase SHP2. *Bioorganic Med. Chem.* **25 (24),** 6479-6485 (2017).

16 Cheng, Y., & Prusoff W.H. Relationship between the inhibition constant (K_I) and the concentration of inhibitor which causes 50 per cent inhibition (I_{50}) of an enzymatic reaction. *Biochem. Pharmacol.* **22,** 3099–3108 (1973).

17 Horatscheck, A. *et al.* Benzoylphosphonate-based photoactive phosphopeptide mimetics for modulation of protein tyrosine phosphatases and highly specific labeling of SH2 domains. *Angew. Chem. Int. Ed.* **51,** 9441-9447 (2012).

18 Molina, D. M. *et al.* Monitoring drug target engagement in cells and tissues using the cellular thermal shift assay. *Science* **341,** 84-87 (2013).

19 Schneider, C. A., Rasband, W. S. & Eliceiri, K. W. NIH Image to ImageJ: 25 years of image analysis. *Nat. Methods* **9,** 671-675 (2012).

20 Walker, S., Nelson, E. & Frank, D. STAT5 represses BCL6 expression by binding to a regulatory region frequently mutated in lymphomas. *Oncogene* **26,** 224-233 (2007).

21 Almqvist, H. *et al.* CETSA screening identifies known and novel thymidylate synthase inhibitors and slow intracellular activation of 5-fluorouracil. *Nat. Commun.* **7,** 11040 (2016).

22 Krutzik, P. O. & Nolan, G. P. Fluorescent cell barcoding in flow cytometry allows high-throughput drug screening and signaling profiling. *Nat. Methods* **3,** 361 (2006).

23 Sun, H., Panicker, R. C., & Yao, S. Q. Activity based fingerprinting of proteases using FRET peptides. *J. Pept. Sci.* **88 (2),** 141-149 (2007).

24 Wagner, S., Schütz, A., & Rademann, J. Light-switched inhibitors of protein tyrosine phosphatase PTP1B based on phosphonocarbonyl phenylalanine as photoactive phosphotyrosine mimetic. *Bioorganic Med. Chem.* **23 (12),** 2839-2847 (2015).
