# Supplementary material for: The transcription factor STAT5 catalyzes Mannich ligation reactions yielding inhibitors of leukemic cell proliferation
Source: Nat Commun. 2019 Jan 8;10:66. doi: 10.1038/s41467-018-07923-2 (PMC6325109; doi:10.1038/s41467-018-07923-2)
Supplement: Supplementary file 3 — Reporting Summary [file 41467_2018_7923_MOESM3_ESM.pdf]

nature research

Corresponding author(s): Prof. Dr. Jörg Rademann

Reporting Summary

Nature Research wishes to improve the reproducibility of the work that we publish. This form provides structure for consistency and transparency in reporting. For further information on Nature Research policies, see Authors & Referees and the Editorial Policy Checklist.

Statistical parameters

When statistical analyses are reported, confirm that the following items are present in the relevant location (e.g. figure legend, table legend, main text, or Methods section).

n/a	Confirmed	
☐	☒	The exact sample size (n) for each experimental group/condition, given as a discrete number and unit of measurement
☐	☒	An indication of whether measurements were taken from distinct samples or whether the same sample was measured repeatedly
☐	☒	The statistical test(s) used AND whether they are one- or two-sided<br>*Only common tests should be described solely by name; describe more complex techniques in the Methods section.*
☒	☐	A description of all covariates tested
☒	☐	A description of any assumptions or corrections, such as tests of normality and adjustment for multiple comparisons
☐	☒	A full description of the statistics including central tendency (e.g. means) or other basic estimates (e.g. regression coefficient) AND variation (e.g. standard deviation) or associated estimates of uncertainty (e.g. confidence intervals)
☐	☒	For null hypothesis testing, the test statistic (e.g. F, t, r) with confidence intervals, effect sizes, degrees of freedom and P value noted<br>*Give P values as exact values whenever suitable.*
☒	☐	For Bayesian analysis, information on the choice of priors and Markov chain Monte Carlo settings
☒	☐	For hierarchical and complex designs, identification of the appropriate level for tests and full reporting of outcomes
☒	☐	Estimates of effect sizes (e.g. Cohen's d, Pearson's r), indicating how they were calculated
☐	☒	Clearly defined error bars<br>*State explicitly what error bars represent (e.g. SD, SE, CI)*

Our web collection on statistics for biologists may be useful.

Software and code

Policy information about availability of computer code

Data collection	Magellan™ for data collection in Tecan, BD CellQuest™ Pro Software for data acquisition in FACScan Flow Cytometer, Autodock Vina and Sybyl8.1 for molecular docking, 6200 series TOF/6500 series Q-TOF/LC-MS, Syngene Pxi for gel documentation and blot visualization, S1000™ Biorad Thermal Cycler for cDNA synthesis, CETSA and ITDRF, Roche Light-Cycler 480 Real-Time PCR System for RT-PCR and TSA.
Data analysis	Graph Prism 5, Flowjo software, PyMol 1.7.4.4, MGLTools 1.5.6, BINANA and LigandScout 4.2 for molecular docking analysis, Agilent MassHunter Workstation for Q-TOF/LC-MS analysis, GeneSys software for western blotting.

For manuscripts utilizing custom algorithms or software that are central to the research but not yet described in published literature, software must be made available to editors/reviewers upon request. We strongly encourage code deposition in a community repository (e.g. GitHub). See the Nature Research guidelines for submitting code & software for further information.

Data

Policy information about availability of data

All manuscripts must include a data availability statement. This statement should provide the following information, where applicable:

- Accession codes, unique identifiers, or web links for publicly available datasets
- A list of figures that have associated raw data
- A description of any restrictions on data availability

The datasets generated and analyzed in the current study are available from the corresponding author upon reasonable request.

Field-specific reporting

Please select the best fit for your research. If you are not sure, read the appropriate sections before making your selection.

☒ Life sciences ☐ Behavioural & social sciences ☐ Ecological, evolutionary & environmental sciences

For a reference copy of the document with all sections, see nature.com/authors/policies/ReportingSummary-flat.pdf

Life sciences study design

All studies must disclose on these points even when the disclosure is negative.

Sample size	Sample sizes were determined by triplicate samples or more (n≥ 3) for comparisons between one or multiple groups, followed by the statistical analysis.The group size, i.e. the number of experimental animals per group is calculated using a statistical program (e.g., Sigma Stat, SAS) for an ANOVA evaluation based on the following parameters: difference of the target size tumor area, variance of the target size and number of groups. The target size is the tumor area, which is calculated after measuring the length and width of the implanted tumors. Based on the variance of the test parameter tumor growth (standard deviation), n=6 animals per group were used to secure statistical significance.
Data exclusions	No data were excluded from the analyses.
Replication	The data were reliably reproduced in repeated experiments. The animal data were collected and analyzed from enough mice for each group.
Randomization	Animals were randomized with Microsoft Excel into treatment or control groups. For mouse xenograft assays (Figure 6f), the mice were randomized and then treated with or without compound 16.
Blinding	The investigators were not blinded. Investigators were not blind to group allocation during data collection and analysis because the absolute values of tumor volume and tumor weight were recorded for statistic analysis.

Reporting for specific materials, systems and methods

Materials & experimental systems

n/a	Involved in the study	
☒	☐	Unique biological materials
☐	☒	Antibodies
☐	☒	Eukaryotic cell lines
☒	☐	Palaeontology
☐	☒	Animals and other organisms
☒	☐	Human research participants

Methods

n/a	Involved in the study	
☒	☐	ChIP-seq
☐	☒	Flow cytometry
☒	☐	MRI-based neuroimaging

Antibodies

Antibodies used	Please see antibodies and reagents in the method section for the species the antibodies were derived from, the source of the antibodies and the clone numbers if applicable. Below the catalog numbers (where applicable) are listed: anti-STAT5a (C-6, cat. sc271542) and STAT5b (G-2, cat. sc-1656), anti-STAT3 (C-20, cat. sc-482), anti-phospho-STAT3 (Tyr 705) (cat. sc-7993), anti-STAT5 (C-17, cat. sc-835) and anti-Bcl-xL (H-5, cat. sc-8392) were obtained from Santa Cruz Biotechnology (Santa Cruz, CA). Antibodies recognizing Cyclin D1 (92G2, cat. 2978s), FLT3 (8F2, cat. 3462s), phospho-STAT5 (Tyr694) (cat. 9351), phospho-FLT3 Tyr591 (cat. 3461s) were obtained from Cell Signaling Technology (Danvers, MA). Anti-Pim1 (M08, clone 1C10, cat. H00005292-M08) was purchased from Abnova (Taiwan), anti-Beta actin (cat. ab8227) was from Abcam (Cambridge, MA), anti Biotin–Peroxidase antibody (cat. A4541) was obtained from Sigma Aldrich (Taufkirchen,

Germany) and anti-CIS (cat. PA5-27128) was from Thermo Fisher Scientific (Braunschweig, Germany). For phospho flow cytometry experiments, PE-Cy™7 mouse anti-Stat5 (pY694) (clone 47, cat. 560117) and anti-mouse IgG1 monoclonal antibody (clone A85-1, cat. 550083) were from BD Biosciences. Antibodies were validated by the manufacturers.Recombinant mouse IL3 protein (cat. PMC0034) was purchased from Thermo Fisher Scientific and midostaurin hydrate (PKC412, cat. M1323) was from Sigma-Aldrich.

Validation

Antibodies were validated by the manufacturers.

Eukaryotic cell lines

Policy information about cell lines

Cell line source(s)

MV-4-11 (ATCC® CRL-9591™) and K562 (ATCC® CCL-243™) suspension cell lines; COS-7 (COS-7 ATCC ® CRL-1651™), MDA-MB-231 ((ATCC® HTB-26™), HT29 (ATCC ® HTB-38™) and HeLa (ATCC® CCL-2™) adherent cell lines were purchased from ATCC. BaF3 and BaF3/FLT3-ITD cells were kind gifts from Prof. Dr. Carol Stocking from University Hospital Hamburg.

Authentication

A Certificate of Analysis was provided by ATCC.

Mycoplasma contamination

All cell lines were tested and were mycoplasma-free using MycoAlert Mycoplasma Detection Kit (Lonza).

Commonly misidentified lines
(See ICLAC register)

No commonly misidentified cell lines were used in this study.

Animals and other organisms

Policy information about studies involving animals; ARRIVE guidelines recommended for reporting animal research

Laboratory animals

6 weeks old NSG mice (NOD/Shi-scid/IL-2Rγnull) female mice with average body weight of 22 grams were used for the experiments in Figure 6e-f.All animal experiments were carried out in accordance with the United Kingdom Coordinating Committee on Cancer Research Regulations for the Welfare of Animals and in accordance with the German Animal Protection Law approved by the local responsible authorities, Berlin, Germany.

Wild animals

N/A

Field-collected samples

N/A

Flow Cytometry

Plots

Confirm that:

☒ The axis labels state the marker and fluorochrome used (e.g. CD4-FITC).

☒ The axis scales are clearly visible. Include numbers along axes only for bottom left plot of group (a 'group' is an analysis of identical markers).

☒ All plots are contour plots with outliers or pseudocolor plots.

☒ A numerical value for number of cells or percentage (with statistics) is provided.

Methodology

Sample preparation

Figure 5e5(i) , Supplementary Figure 6a and 7a: Cell apoptosis was determined using Annexin V staining. BaF3/FLT3-ITD or K562 suspension cells were plated at 0.2x10^6 per well in 6-well plates and incubated with serial concentration of tested compounds for 48 h. Cells were washed twice in ice-cold PBS resuspended in 1x binding buffer (10 mM Hepes (pH 7.4), 140 mM NaCl and 2.5 mM CaCl2) to a final concentration of 1x 10^6 cells/ml. Subsequently, FITC Annexin V solution (5 µl) was added to 100 µl of the cell suspension. The mixture was incubated for 30 min at room temperature and washed in 1x Binding Buffer and again resuspended in 200 µl of 1x Binding buffer. Propidium iodide staining solution (5 µl, Sigma) was added shortly before analysis on a FACScan Flow Cytometer (Becton-Dickinson, San Jose, CA). Data interpretation was done using the Flowjo software (Treestar, Inc., San Carlos, CA).

Figure 5e (ii) and Supplementary Figure 7b: BaF3/ FLT3-ITD or K562 cells were seeded at 0.2 x10^6 cells/ml per well in 6-well plates overnight and incubated with serial concentration of tested compounds for 6 h. A total amount of 10^6 cells were collected and washed twice in 1xPBS and resuspended in 100 µl cytofix/cytoperm solution (BD Biosciences) at 4 ºC. After 20 min, cells were washed twice with BD Perm/Wash buffer solution and incubated with 20 µl of specific fluorochrome (PE) conjugated monoclonal antibody anti-p-STAT5 (Phosflow™ PE-Cy™7 mouse anti-Stat5 (pY694) (BD Biosciences) at 4 ºC. After 30 min, the cells were washed twice and analyzed by flow cytometry. For isotype control, the cells were incubated with 2 µL of PE conjugated rat anti-mouse IgG1 monoclonal antibody (BD Biosciences) at 4 ºC for 30 min and washed twice in before being analyzed on a FACScan Flow Cytometer (Becton-Dickinson, San Jose, CA). Data interpretation was done using the Flowjo software (Treestar, Inc., San Carlos, CA).

Detail sample preparation is provided in the Supplementary Methods section.

Instrument	FACScan Flow Cytometer (Becton-Dickinson, San Jose, CA).
Software	BD CellQuest™ Pro Software for data acquisition and Flowjo software for analysis.
Cell population abundance	At least 10.000 events were acquired per sample and cell sorting is not employed.
Gating strategy	Using the FSC/SSC gating, debris was removed by gating on the main cell population. All samples were then FSC-A and SSC-A gated, followed by FSC-A/FSC-H gating to select singlet cells.Positivity threshold for each cell line was defined on the basis of mock-treated sample. Identical positivity threshold was applied to all samples within cell line.

☒ Tick this box to confirm that a figure exemplifying the gating strategy is provided in the Supplementary Information.
